# Delineation of Taxonomic Species within Complex of Species: *Aeromonas media* and Related Species as a Test Case

**DOI:** 10.3389/fmicb.2017.00621

**Published:** 2017-04-18

**Authors:** Emilie Talagrand-Reboul, Frédéric Roger, Jean-Luc Kimper, Sophie M. Colston, Joerg Graf, Fadua Latif-Eugenín, Maria José Figueras, Fabienne Petit, Hélène Marchandin, Estelle Jumas-Bilak, Brigitte Lamy

**Affiliations:** ^1^Équipe Pathogènes Hydriques Santé Environnements, UMR 5569 HSM, Université de MontpellierMontpellier, France; ^2^Département d'Hygiène Hospitalière, CHRU de MontpellierMontpellier, France; ^3^Department of Molecular and Cell Biology, University of ConnecticutStorrs, CT, USA; ^4^Unidad de Microbiologia, Facultad de Medicina y Ciencias de la Salud, IISPV, Universidad Rovira i VirgiliReus, Spain; ^5^Normandie Univ, UNIROUEN, UNICAEN, Centre National de la Recherche Scientifique, M2CRouen, France; ^6^Sorbonne Universités, UPMC, Centre National de la Recherche Scientifique, EPHE, UMR 7619 METISParis, France; ^7^Département de Bactériologie, CHRU de MontpellierMontpellier, France; ^8^Département de Bactériologie, CHU de NiceNice, France

**Keywords:** population study, integrative taxonomy, *Aeromonas*, speciation, complex of species, taxogenomics, phylogeny, recombination

## Abstract

*Aeromonas media* is an opportunistic pathogen for human and animals mainly found in aquatic habitats and which has been noted for significant genomic and phenotypic heterogeneities. We aimed to better understand the population structure and diversity of strains currently affiliated to *A. media* and the related species *A. rivipollensis*. Forty-one strains were included in a population study integrating, multilocus genetics, phylogenetics, comparative genomics, as well as phenotypics, lifestyle, and evolutionary features. Sixteen gene-based multilocus phylogeny delineated three clades. Clades corresponded to different genomic groups or genomospecies defined by phylogenomic metrics ANI (average nucleotide identity) and *is*DDH (*in silico* DNA-DNA hybridization) on 14 whole genome sequences. DL-lactate utilization, cefoxitin susceptibility, nucleotide signatures, ribosomal multi-operon diversity, and differences in relative effect of recombination and mutation (i.e., in evolution mode) distinguished the two species *Aeromonas media* and *Aeromonas rivipollensis*. The description of these two species was emended accordingly. The genome metrics and comparative genomics suggested that a third clade is a distinct genomospecies. Beside the species delineation, genetic and genomic data analysis provided a more comprehensive knowledge of the cladogenesis determinants at the root and inside *A. media* species complex among aeromonads. Particular lifestyles and phenotypes as well as major differences in evolution modes may represent putative factors associated with lineage emergence and speciation within the *A. media* complex. Finally, the integrative and populational approach presented in this study is considered broadly in order to conciliate the delineation of taxonomic species and the population structure in bacterial genera organized in species complexes.

## Introduction

The genus *Aeromonas* groups ubiquitous bacteria mainly found in aquatic habitats. Among the 30 taxonomic species currently described in the genus, half were characterized since the latest edition of the Bergey's Manual of Systematic Bacteriology in 2005 (Martin-Carnahan and Joseph, 2005). Taxonomy in the genus is subject to controversies leading to several reclassifications (Beaz-Hidalgo et al., 2013). The main reason is the organization of *Aeromonas* in several species complexes, heterogeneous groups of related but genetically distinct strains. Taxonomic focus on a species complex generally leads to the delineation of new species from the most homogeneous groups inside the complex. Consequently, an increasing number of new species are described in the genus, but some of these descriptions lead to subsequent controversies about their delineation and robustness.

*Aeromonas media* is one typical example among others. Heterogeneity in the complex *Aeromonas media* was recognized early after its description because it was split in two DNA-DNA hybridization groups (HG), HG5A and HG5B represented by strains Popoff 233 and 239, respectively (Altwegg et al., 1990). The type strain *A. media* CECT 4232^T^ groups into HG5B (Altwegg et al., 1990). *A. media* has been described in river freshwater (Allen et al., 1983) and was then found in sewage water, activated sludge, drinking water, animals and human (Picao et al., 2008; Pablos et al., 2009; Figueira et al., 2011; Roger et al., 2012a). *A. media* acts as an opportunistic emerging pathogen causing a wide spectrum of diseases in human and animals (Beaz-Hidalgo et al., 2010; Figueras and Beaz-Hidalgo, 2015). It causes skin ulcers in fish and diarrhea in human, but with lower prevalence than other clinically relevant *Aeromonas* species (Singh, 2000; Parker and Shaw, 2011; Figueras and Beaz-Hidalgo, 2015).

The intra-species heterogeneity of *A. media* was confirmed by 16S rRNA gene, *rpoB* and *gyrB* sequencing that showed that strains distributed into two subgroups corresponding to the HG subgroups (Küpfer et al., 2006). Considering phenotype, every HG5B strain but no HG5A strains utilizes DL-lactate (Altwegg et al., 1990). A new species related to *A. media, A. rivipollensis* was recently described (Marti and Balcázar, 2015; Oren and Garrity, 2016) but was not positioned into any of the HG subgroups. In addition, *A. media* displays distinctive genetic features compared to other *Aeromonas* species: (i) a genetic polymorphism showed by 7 gene-multilocus sequence analysis (MLSA) higher than that of any other species within the genus (5.8% vs. a mean of 2.5% for other species) (Roger et al., 2012a), (ii) a high heterogeneity in the tRNA genes intergenic spacers (Laganowska and Kaznowski, 2005), (iii) one of the highest rate of intragenomic heterogeneity among *rrn* operons (Alperi et al., 2008; Roger et al., 2012b), and (iv) a strikingly high diversity of *rrn* chromosomal distribution (Roger et al., 2012b). However, all above studies were not designed for assessing the heterogeneity in *A. media* and included a rather low number of *A. media* strains. HG subgroups and the heterogeneity observed suggested that *A. media* and the related species *A. rivipollensis* formed a species complex (SC) referred hereafter as “*Media* SC.”

In such a context, population studies are powerful means to investigate heterogeneity within a complex of species (Vandamme and Dawyndt, 2011). This work aimed to perform an integrative approach to structure a population of 41 strains (14 whole genome sequences) from different habitats and geographical regions affiliated to *Media* SC. In the context of species complex, recombination events have been particularly taken into consideration. Beyond the population-based reappraisal of the phylotaxonomy, *Media* SC is taken as an outstanding test case to discuss the conflicts between species delineation and population structure in bacteria, and to highlight how the population studies can reinforce knowledge in taxonomy.

## Materials and methods

### Bacterial strains, culture conditions, and DNA extraction

A total of 40 *A. media* previously identified by *gyrB* sequencing were analyzed (Table 1), including the type strain of *A. media* (CECT 4232^T^), and reference strains for HG5A (*A. caviae* LMG 13459) and HG5B (*A. caviae* LMG 13464). Fifty-four strains belonging to 30 other species of *Aeromonas* were also studied, including the type strain of every species with validated name of which the new species *A. rivipollensis* Marti and Balcazar 2016 (LMG 26323^T^) (Marti and Balcázar, 2015; Oren and Garrity, 2016). Strains were grown on Trypticase Soy Agar at 35°C for 16–24 h, and genomic DNA were extracted using the MasterPure™ DNA Purification Kit (Epicentre, USA). All the bacterial experiments were performed at Biosafety Level 2.

**Table 1:** **Characteristics of the 41 strains affiliated to *Media* species complex on the basis of *gyrB* sequences analyzed in this study**.

**Strain**	**Clade in MLP**	**Genome accession number**	**Sample**	**Origin**	**Country (Date of isolation)**	**References**
*A. media* CCM 4242	A		River water	Environment	Czech republic (1991)	Sedlácek et al., 1994
*A. rivipollensis* LMG 26313^T^	A		River biofilm (output WWTP)	Environment	Spain (2010)	Marti and Balcázar, 2015
LL6-17C	A		River water (output WWTP)	Environment	Spain (2012)	This study
SS1-15D	A		WWTP	Environment	Spain (2012)	This study
CR4.2-17C	A		River water	Environment	Spain (2012)	This study
*A. hydrophila* 4AK4^a^	A	PRJNA210524^b^	Raw sewage	Environment	NA (NA)	Gao et al., 2013
*A. caviae* LMG 13459 = Popoff 239	A	PRJEB12346^b^	Fish (infected)	Animal	France (1967-1974)	Popoff and Véron, 1976
*A. media* AK202	A	PRJEB12343^b^	Snail	Animal	France (1995)	Roger et al., 2012b
AK208	A		Snail	Animal	France (1995)	This study
AK210	A		Snail	Animal	France (1995)	This study
*A. hydrophila* AH31	A		Crab	Animal	Norway (1998)	Granum et al., 1998
R1	A		Salmon	Animal	Spain (2007)	This study
R100	A		Trout	Animal	Spain (2007)	This study
417-16G	A		Mussel	Animal	Spain (2011)	This study
M2C164 = M2C A28	A		Copepod	Animal	France (2012)	This study
M2C185 = M2C A42	A		Copepod	Animal	France (2012)	This study
M2C205 = M2C A47	A		Copepod	Animal	France (2012)	This study
*A. media* 76C	A	PRJEB8966^b^	Human diarrheic stool	Clinical	Spain (1992)	Mosser et al., 2015
BVH17	A		Human urine (healthy carriage)	Clinical	France (2006)	This study
*A. media* BVH40	A	PRJEB8017^b^	Human stool (healthy carriage)	Clinical	France (2006)	Roger et al., 2012a
ADV137a	A		Human respiratory tract (near-drowning)	Clinical	France (2010)	This study
*A. media* CECT 4232^T^ = RM	B	PRJEB7032^b^	River water (fish farm effluent)	Environment	UK (1980)	Allen et al., 1983
*A. media* CECT 4234 = P1E	B		River water (fish farm pond)	Environment	UK (1981)	Allen et al., 1983
T41-3	B		Soil	Environment	Spain (2010)	This study
T41-7	B		Soil	Environment	Spain (2010)	This study
RT10-17Ga	B		River water	Environment	Spain (2012)	This study
RT11-17Ga	B		River water	Environment	Spain (2012)	This study
LL4.4-18D	B		River water (input WWTP)	Environment	Spain (2012)	This study
SEL1-18A	B		WWTP	Environment	Spain (2012)	This study
CR4-18pb	B		River water	Environment	Spain (2012)	This study
*A. media* ARB13^a^	B	PRJNA260228^c^	River water	Environment	Japan (2013)	Kenzaka et al., 2014
*A. media* ARB20^a^	B	PRJNA260227^c^	River water	Environment	Japan (2013)	Kenzaka et al., 2014
*A. media* WS^a^	B	CP007567.1^c^	Lake water	Environment	China (2003)	Chai et al., 2012
*A. caviae* LMG 13464 = Popoff 233	B	PRJEB12347^b^	Fish (infected)	Animal	France (1967-1974)	Popoff and Véron, 1976
AK207	B		Snail	Animal	France (1995)	This study
*A. media* AK211	B	PRJEB12344^b^	Snail	Animal	France (1995)	Roger et al., 2012a
*Aeromonas sp*. CECT 7111	B	PRJEB12345^b^	Oyster (*Crassostrea gigas*)	Animal	Spain (2002)	Miñana-Galbis et al., 2002
BVH83	B		Human urine (healthy carriage)	Clinical	France (2006)	This study
D84402	B		Human diarrheic stool	Clinical	Spain (2012)	This study
*A. eucrenophila* UTS 15	C	PRJEB12350^b^	Koi carp	Animal	Australia (2001)	Carson et al., 2001
1086C = CECT 8838 = LMG 28708	C	PRJEB12349^b^	Human diarrheic stool	Clinical	Spain (2010)	This study

*Several strains were wrongly labeled at the time of their isolation but all the 41 strains belonged to the Media SC on the basis of gyrB sequences that is robust enough for their affiliation within the genus Aeromonas, as explained in the main text*.

*MLP, Multi-Locus Phylogeny; NA, Not available; WWTP, Waste water treatment plant*.

a *Strains studied only by genetic and genomic analysis*.

b *Obtained from the EMBL Nucleotide Sequence Database*.

c *Obtained from GenBank, National Center for Biotechnology Information*.

### Phenotypic characterization

Methods are given in the Supplementary Taxonomic Sheet.

### Gene amplification and sequencing

The 16S rRNA genes and 16 housekeeping (HK) genes described in three MLSA schemes (*atpD, dnaJ, dnaK, dnaX, gltA, groL, gyrA, gyrB, metG, ppsA, radA, recA, rpoB, rpoD, tsf*, and *zipA*) were amplified using primers and PCR conditions previously described (Carlier et al., 2002; Martinez-Murcia et al., 2011; Martino et al., 2011; Roger et al., 2012b). For strains affiliated to *A. media*, the oligonucleotide zipA-Rmed (5′-CATGTTGATCATGGAGAAGAGC-3′) was designed in this study and used as reverse primer for *zipA* amplification. Amplification products were sequenced using either forward primers (housekeeping genes) or both forward and reverse primers (16S rDNA) on an ABI 3730XL automatic sequencer (Beckman Coulter Genomics, France).

### Phylogenetic analyses

Phylogeny was inferred from gene sequences produced in this study or downloaded from GenBank. Gene sequences were aligned using the Clustal ω2 program within Seaview 4 package (Gouy et al., 2010). PhyML trees were reconstructed for (i) every 16 individual HK locus and 16S rRNA gene (Single Locus Phylogeny, SLP), (ii) the concatenated HK loci (Multi Locus Phylogeny, MLP), (iii) the concatenated translated amino acid sequences, and (iv) for the concatenated codon-aligned nucleotidic HK sequences excluding third position. Maximum likelihood (ML) phylogenetic trees were reconstructed from nucleotidic sequences using a GTR model plus gamma distribution and invariant sites as substitution model, in PhyML v3.1 program. The ML tree was reconstructed from concatenated amino acid sequences using the Jones-Taylor-Thornton (JTT) model (Jones et al., 1992). ML bootstrap supports were calculated after 100 reiterations. Models used were determined to be the most appropriate after Akaike criterion calculation by IQ-TREE at http://iqtree.cibiv.univie.ac.at/.

### Study of genetic recombination

Horizontal gene transfer (HGT) was detected from the concatenated sequences using a polyphasic approach. The program LIAN v3.7, available at http://www.pubmlst.org, was used to calculate the standardized index of association (*I*^s^_A_), to test the null hypothesis of linkage disequilibrium, and to determine mean genetic diversity (H) and genetic diversity at each locus (h). A distance matrix in nexus format was generated from the set of allelic profiles and then used for decomposition analyses with the neighbor-net algorithm available in SplitsTree 4.0 software (Huson and Bryant, 2006). Recombination events were detected using the pairwise homoplasy index test, φw (Bruen et al., 2006), implemented in SplitsTree 4.0 and the RDP v3.44 software package (Martin et al., 2010) with the basic parameters, as described elsewhere (Roger et al., 2012a). Only positive recombination events supported by at least four of the seven methods used in RDP were taken into account.

Five independent runs of ClonalFrame (Didelot and Falush, 2007) with 300,000 iterations were performed on the whole strain population and within specific lineages for the estimation of ρ/θ (relative rate of recombination and mutation) and r/m (relative effect of recombination and mutation). We fixed values of δ, the mean tract length of imported sequence fragment, at 412 bp which is the value inferred during the five runs performed on the whole dataset, and of the mutation rate to the Watterson's moment estimator θW obtained from the whole population or from each lineage population (Chaillou et al., 2013).

### RIMOD (ribosomal multi operon diversity)

For *rrn* numbering and chromosome distribution study, intact genomic DNA was prepared in agarose plugs as previously described (Roger et al., 2012b). Number of *rrn* copies was deduced from the number of I-*Ceu*I-generated fragments. The Pulsed-Field Gel Electrophoresis (PFGE) profiles were visually compared and both shared and distinct DNA fragments were numbered. Size of PFGE bands were measured by comparison with lambda concatemer ladder (New England BioLabs) used as size standard. For studying intragenomic *rrs* heterogeneity, amplification by PCR of a 199 bp fragment overlapping the 16S rRNA gene variable region V3, Temporal Temperature Gradient Gel Electrophoresis (TTGE), and band analysis and sequencing were performed as previously described (Roger et al., 2012b). TTGE bands and profiles were numbered according to Roger et al. (2012b) with increment for previously undescribed bands.

### Genome sequencing and analysis

Ten strains were sequenced at the Microbial Analysis, Resources and Services (MARS) facility at the University of Connecticut (Storrs, USA) with the Illumina MiSeq benchtop sequencer, as described previously (Colston et al., 2014), after preparing libraries from the genomic DNA using NexteraXT DNA sample preparation kit (Illumina, San Diego, CA). Paired Illumina reads were trimmed and assembled into scaffolded contigs using the *de novo* assembler of CLC Genomics Workbench versions 6.0.04 to 7.0.04 (CLC-bio, Aarhus, Denmark) (Supplementary Table 1). Four other whole genome sequences (WGS) were downloaded from public databases on 6 November 2014 (Supplementary Table 1). Genomic contigs and circular genomes were annotated by using the RAST annotation server (Overbeek et al., 2014). Assembled contigs were reconstituted from RAST to generate GenBank files for all genomes using the seqret function of the EMBOSS package (Rice et al., 2000). Homologous translated genes were identified using the program GET_HOMOLOGUES which uses a BLASTP bidirectional best-hit approach with OrthoMCL and COG clustering algorithms (Contreras-Moreira and Vinuesa, 2013). Four clusters of genes were determined from the pangenome analysis: core-genome present in all the genomes, softcore-genome present in 95% (*n* = 13) of the genomes, shell-genome present in ≥3 and <13 genomes and cloud-genome present in ≤ 2 genomes. The parse_pangenome_matrix.pl script was employed to extract the specific conserved genome of selected lineages. Average Nucleotide Identity (ANI) was determined using the Jspecies package 1.2.1 (Richter and Rosselló-Móra, 2009) and default parameters. An estimate of *in silico* DNA-DNA Hybridization (*is*DDH) was made using genome-to-genome distance calculator (GGDC) (Meier-Kolthoff et al., 2013). The contig files were uploaded to the GGDC 2.0 webserver (http://ggdc.dsmz.de/distcalc2.php), and *is*DDH was calculated using formula 2, independent of genome length, as recommended by the authors of GGDC for use with any incomplete genomes. Genome sizes and G + C DNA contents were estimated *in silico* after WGS analysis by RAST.

### Statistics

All qualitative variables, with the exception of the *I*^s^_A_ and ClonalFrame results, were compared using a Chi-squared test, the Fisher's exact test or the Student's test where appropriate; *P*-value ≤ 0.05 was considered significant. All these computations were performed using R project software (http://www.r-project.org). The congruence of results obtained with MLP analysis, PCR-TTGE and PFGE were evaluated with the adjusted Wallace coefficient with 95% confidence interval (CI). Calculations were done using the Comparing Partitions website (www.comparingpartitions.info). Satisfactory convergence of the Markov Chain Monte Carlo, MCMC, in the different runs of ClonalFrame was estimated based on the Gelman-Rubin statistic implemented in the software (Didelot and Falush, 2007).

### Nucleotide sequence and genome accession numbers

The nucleotide sequences determined in this study were deposited in the GenBank database under accession numbers KP400769 to KP401575 and KU756255 to KU756265 (HK), KP717966 to KP718060 and KX553956 to KX553959 (V3 region of the 16S rRNA gene), KT934807 to KT934809 and KU363310 to KU363342 (almost complete 16S rRNA gene). The genomic sequences determined in this study were deposited in the European Nucleotide Archive (ENA) database with the accession numbers referenced in Table 1, and are also available for query and download at http://aeromonasgenomes.uconn.edu.

## Results

### Collection representativeness

The *gyrB* gene was selected as an acceptable marker because it is both accurate enough to discriminate between *A. rivipollensis* and *A. media* strains (*Media* SC sequence similarity ≥94.6%, interspecies similarity ≤ 95.3%), and it is widely used for characterizing *Aeromonas* strains (Supplementary Taxonomic Sheet).

**Figure 1:**
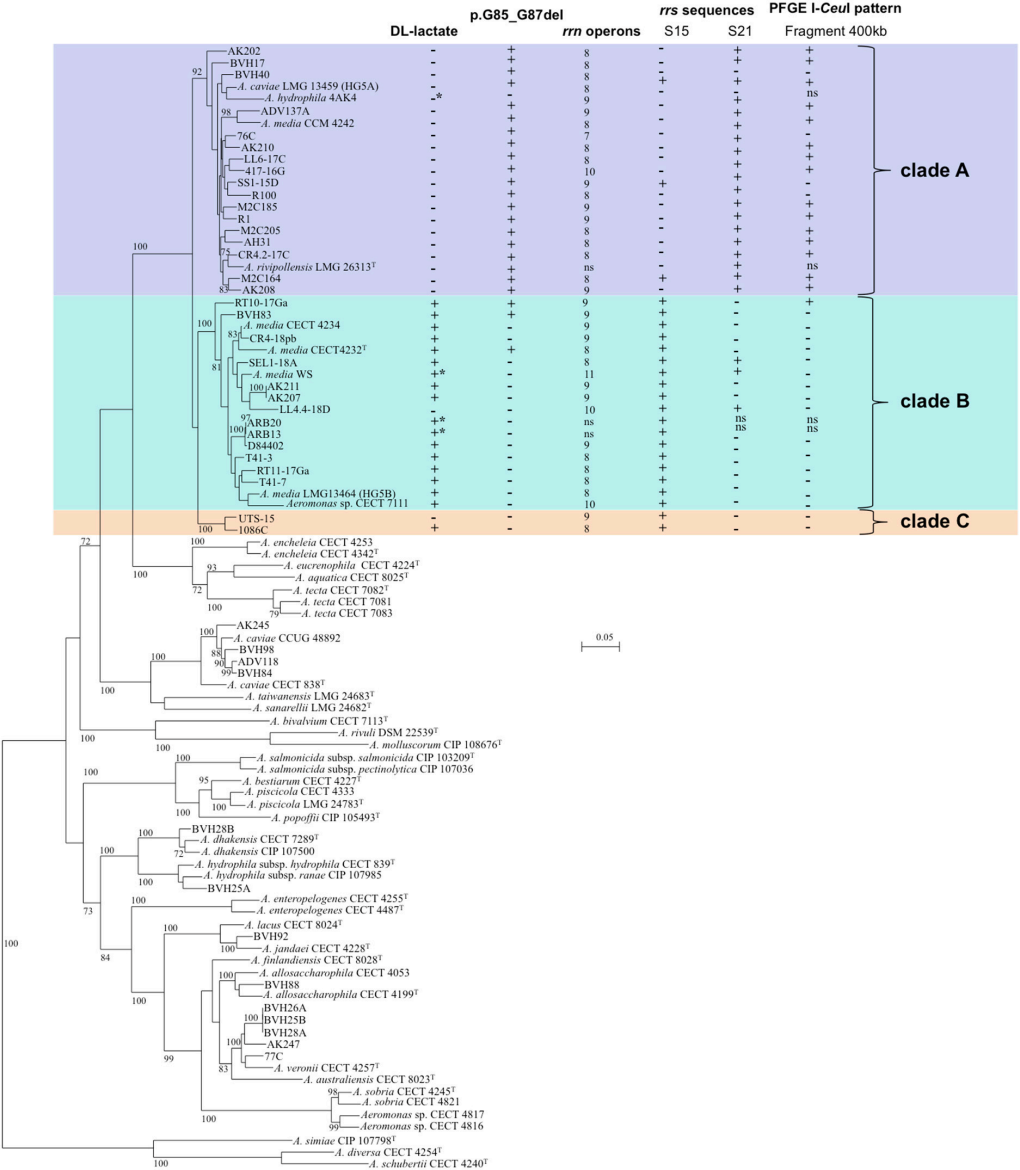
**Unrooted Maximum-Likelihood tree based on concatenated sequences of 16 housekeeping gene fragments (9,427 nt.) from 3 MLST schemes**. The tree shows the phylogenetic structure of the studied *Media* species complex population and the relative placement of strains to other recognized species in the genus. The horizontal lines represent genetic distance, with the scale bar indicating the number of substitutions per nucleotide position. The numbers at the nodes are support values estimated with 100 bootstrap replicates. Only bootstrap values ≥70 are indicated. The following characteristics are indicated for strains affiliated to *A. media* from left to right: (i) DL-lactate utilization, (ii) presence/absence of a 3 amino acid deletion (p.G85_G87del^†^), corresponding to a deletion of 9 nucleotides (c.3357226_3357234del^††^), (iii) number of *rrn* operons, (iv) presence/absence of 16S rRNA gene sequences S15 and/or S21 (corresponding to PCR-TTGE bands No. 15 and/or No. 21) revealed either by analysis of WGS or PCR-TTGE pattern, (v) presence/absence of DNA fragment of about 400 kb in PFGE I-*Ceu*I pattern. The type strain of *Aeromonas fluvialis* was not included in the phylogenetic tree either because we failed to amplify the locus *ppsA*. This species did not group with *Media* complex in a 7 gene (*atpD, dnaJ, dnaX, gyrA, gyrB, recA, rpoD*)-based tree. ^*^DL-lactate utilization or absence inferred from genomic analysis; +, positive; −, negative; ns, not studied. ^†^position on the chaperone protein DnaJ sequence of *A. hydrophila* subsp. *hydrophila* ATCC 7966^T^ (GenBank ABK39448.1). ^††^position on the circular chromosome sequence of *A. hydrophila* subsp. *hydrophila* ATCC 7966^T^ (NCBI Reference Sequence NC_008570.1).

The *gyrB* sequence of *A. media* CECT 4232^T^ (JN829508, 780 bp) used as query for Megablast analysis detected 125 sequences in NCBI database (30 November 2014, cut-off values of 96% for identity and 50% for query-cover, excluding uncultivated and redundant entry) including 113 strains for which isolation source was known. These 113 strains have been considered for habitat, distribution and pathogenicity. The strains were mainly from environmental (58%) or animal non-human (41%) origin, and 1% of strains were from human origin, a distribution roughly similar to that of the population we studied: 44% of environmental strains, 39% of animal non-human strains and 17% of human clinical strains (Table 1). Redundancy in our population was limited to 2 pairs of strains, including one pair from snails of the same husbandry (AK207 and AK211) and one pair from river water in Japan (ARB13 and ARB20). However, all these strains except ARB13 and ARB20 differed in their sequences as showed in the MLP analysis below (Figure 1). The collection used in this study was representative of the known lifestyles of members of *Media* SC and presented low redundancy.

### Multilocus phylogeny in the genus *Aeromonas*

Phylogenetic relationships among studied strains and in the genus *Aeromonas* are shown on the 16 gene concatenated sequence-based tree (Figure 1). The concatenated sequence allowed the reconstruction of a robust MLP of the genus, and each species formed an independent lineage. The SLP showed lower bootstrap values compared to the multi-locus tree. Phylogeny excluding the third position of codons and protein-based phylogeny displayed lower bootstrap values indicating the low level of homoplasy in the data.

The 40 *A. media* strains and the type strain of *A. rivipollensis* were clustered in a robust MLP lineage (bootstrap value 100%), corresponding to *Media* SC, clearly distinct from other *Aeromonas* species, and contained 3 distinct clades (bootstrap values ≥92%). The clade A contained 21 strains, including the HG5A reference strain LMG 13459 and the type strain *A. rivipollensis* LMG 26323^T^ (Figure 1). The clade B grouped 18 strains, including the HG5B reference strain LMG 13464 and the type strain *A. media* CECT 4232^T^, and the clade C contained 2 strains.

The mean genetic diversity (H) among strains was similar in the 2 main clades (0.9702 and 0.9085 for clades A and B, respectively) (Table 2). The rate of polymorphic sites at each locus was not different between the clades A and B (*P*-value = 0.1). However, on the basis of ClonalFrame analysis, strains in clade A were affected by more mutation events between isolates and their most recent common ancestor than those in clade B, 55 and 3, respectively (Table 2). Some mutations were clade-specific. A deletion of 3 consecutive amino acids (G-F-G), pG85_G87del (*A. hydrophila* subsp. *hydrophila* ATCC 7966^T^ numbering) was detected in the sequence of the chaperone protein DnaJ. This deletion was present in all strains of the clade A with the exception of the strain 4AK4, and in only three strains belonging to clade B (Figure 1; *P*-value < 0.0001). In addition, three specific positions in the aligned *gyrB* sequences (508, 609, and 627) distinguished the 3 clades.

**Table 2:** **Genetic diversity, phylogenetic discrepancies in single locus phylogeny (SLP) analysis and ClonalFrame analysis of bacteria in the *Media* SC according to the population structure observed in multi-locus phylogeny analysis**.

		**Clade A (*n* = 21)**				**Clade B (*n* = 18)**				**Clades A, B, and C (*n* = 41)**		
**Standardized index of association (*I*^s^_A_)**		**0.019 (*P*-value = 0.005)**				**0.310 (*P*-value < 0.0001)**				**0.222 (*P*-value < 0.0001)**		
**Mean genetic diversity (H)**		**0.9702 ± 0.0172**				**0.9085 ± 0.0132**				**0.9730 ± 0.0053**		
		**No of alleles**	**Poly-morphic sites %**	**h, genetic diversity**	**Strains with phylogenetic discrepancies in SLP (most closely related clade)**	**No of alleles**	**Poly-morphic sites %**	**h, genetic diversity**	**Strains with phylogenetic discrepancies in SLP (most closely related clade)**	**No of alleles**	**Polymorphic sites (%)**	**h, genetic diversity**
**Genetic diversity and phylogenetic discrepancies at each locus**	*atpD* (501 bp)	18	7	0.9810	AH31 (clade *A. encheleia*)	10	2	0.9020	−	30	8	0.9768
	*dnaJ* (785–794 bp)	20	6	0.9952	−	13	7	0.9477	BVH83, CECT 4232^T^, RT10-17Ga, CECT 7111, LL4.4-18D (clade A)	34	13	0.9866
	*dnaK* (812 bp)	20	5	0.9905	−	12	16	0.9739	−	34	19	0.9902
	*dnaX* (496 bp)	20	12	0.9952	AK202 (clade *A. caviae*)	12	9	0.9150	−	34	16	0.9829
	*gltA* (433 bp)	19	10	0.9905	LMG 26323 (clade B)	8	3	0.7516	−	29	12	0.9512
	*groL* (510 bp)	18	7	0.9810	−	12	4	0.9346	−	31	11	0.9817
	*gyrA* (469 bp)	8	6	0.7190	R100 (clade *A. caviae*)	12	3	0.9346	−	22	8	0.9159
	*gyrB* (753 bp)	21	7	1.0000	−	12	6	0.9346	−	35	12	0.9878
	*metG* (504 bp)	21	7	1.0000	−	10	8	0.8954	−	33	12	0.9805
	*ppsA* (537 bp)	21	14	1.0000	−	12	11	0.9346	−	35	19	0.9878
	*radA* (405 bp)	20	12	0.9952	AK202 (clade B)	10	3	0.9150	−	32	14	0.9829
	*recA* (598 bp)	19	7	0.9905	−	10	6	0.9216	LL4.4-18D (clade A)	31	11	0.9829
	*rpoB* (426 bp)	16	3	0.9667	−	8	4	0.8301	−	23	6	0.9415
	*rpoD* (503 bp)	19	8	0.9905	−	11	5	0.9085	−	32	12	0.9805
	*tsf* (686 bp)	14	4	0.9381	AK202, BVH17 (clade B)	9	4	0.8889	RT10-17Ga^*^ (clade A)	24	7	0.9512
	*zipA* (423-438 bp)	19	29	0.9905	ADV137a (clade *A. popoffii*/ *A.bestiarum*/ *A. piscicola*); CCM 4242 (clade *A. veronii*)	12	22	0.9477	LL4.4-18D^*^, AK207^*^, AK211^*^ (clade A); CECT 4232^T^ (clade *A. enteropelogenes*)	33	34	0.9878
**Clonal-Frame analysis**	Mutation events^†^	55				3				9		
	Recombination events^†^	10				33				56		
	Substitutions introduced by recombination^†^	95				126				216		
	No of sites without recombination^†^	7364 (83.1% of the sequence length)				2880 (32.5% of the sequence length)				1744 (19.7% of the sequence length)		
	ρ/θ^‡^	0.097 (0.094–0.099)				9.4 (9.4–9.4)				6.5 (6.5–6.5)		
	r/m^‡^	0.89 (0.87–0.90)				37 (36–40)				23 (23–24)		

ρ/θ, relative rate of recombination and mutation;

r/m, relative effect of recombination and mutation;

* *Phylogenetic discrepancy without horizontal gene transfer event detected by RDP software*.

† *Measured between each isolate of each lineage (or the whole population) and their most recent common ancestor*.

‡ *The numbers in brackets are 95% confidence intervals*.

### Recombination rate and horizontal gene transfer in *Media* SC

**Figure 2:**
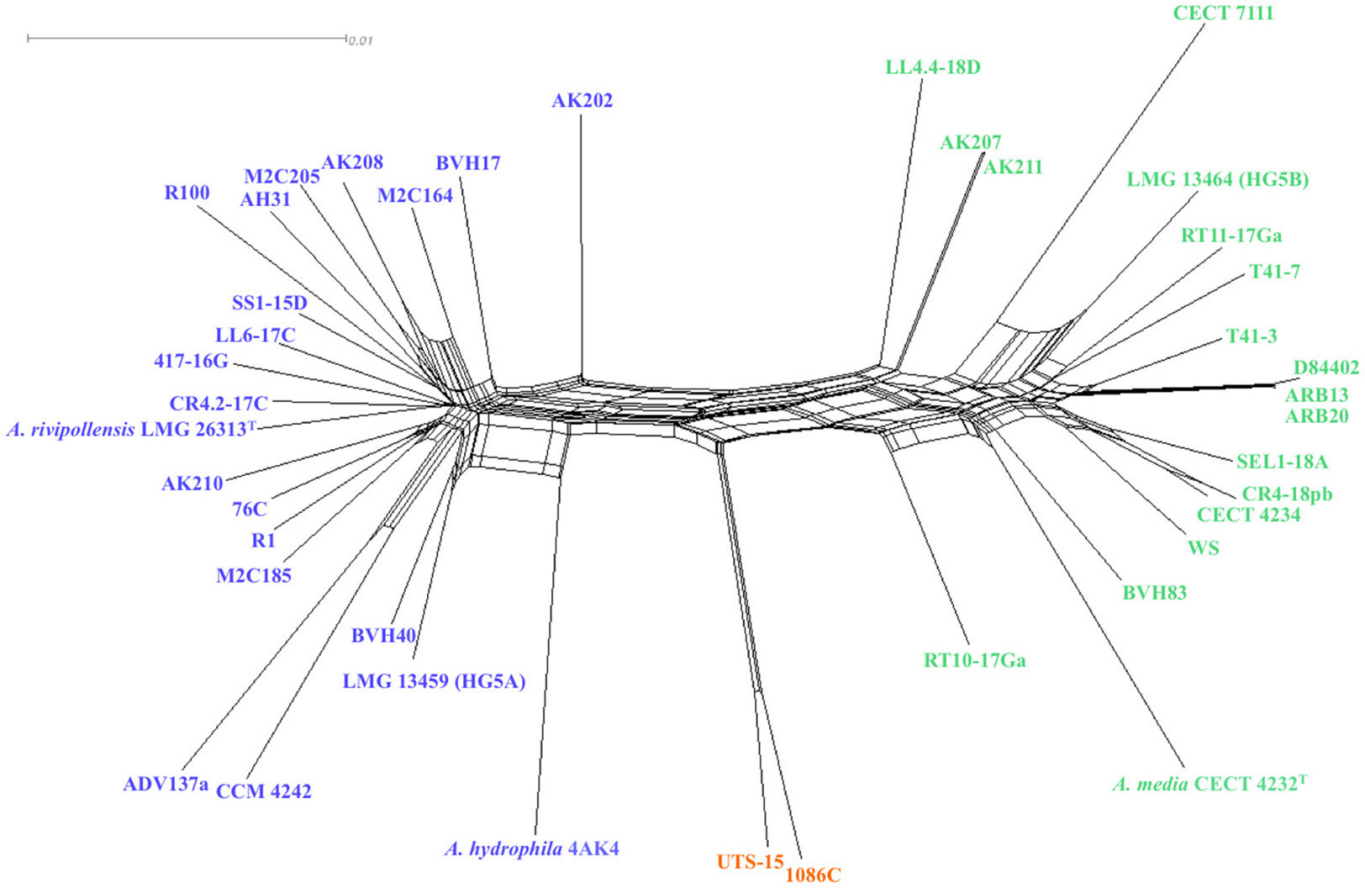
**Neighbor-networks graph based on the concatenated sequences of the 16 housekeeping gene fragments (8,865 nucleotides), showing putative recombination events between 41 strains affiliated to *Media* species complex**. A network-like graph indicates recombination events.

Homoplasy index φW test showed evidence for recombination between clades and within every clade (*P*-value < 0.0001), as confirmed by the interconnected network generated by the Neighbor-Net analysis (Figure 2). However, the network showed clusters congruent with the 3 clades defined in MLP confirming that recombination events were not frequent enough to blur the phylogenetic signal, and that recombination events were more frequent within clades.

Differences in relative branching order among trees reconstructed by HK SLP were observed for 7 strains belonging to clade B and 3 strains of clade A (*P*-value = 0.2) (Table 2). These phylogenetic discrepancies occurred in 6 out of the 16 SLP trees, and this suggested occurrence of HGT events (Table 2) that were further studied by RDP analysis. Twenty-six strains (8 out of the 21 clade A strains and all clades B strains) were detected by RDP for potential recombinant sequences that resulted from at least 16 HGT events spanning from one to three genes. All genes were affected by HGT events, with the exception of *atpD, groL*, and *gyrB* loci. Most strains were affected by one or two events (12 and 9 strains, respectively). Nine events could not be linked to parental sequences, suggesting that transfers occurred from strains that are not represented in our collection.

The *I*^s^_A_ values (0.222, Table 2) supported that *Media* SC displays an overall clonal population structure. However, it appeared that *I*^s^_A_ values associated with clades A and B were highly heterogenic (0.019 and 0.310, respectively, Table 2), and this suggested different trends of population structure without any possibility to draw a clear conclusion, possibly because the scores for the mean genetic diversity for the total population and inside each clade were high (*H* > 0.9, Table 2). Meanwhile, the ClonalFrame analysis, an approach that takes into account the nucleotide sequences and not only the allelic profiles, showed that the number of recombination events detected between isolates of each lineage and their most recent common ancestor was higher in the clade B than in clade A (33 vs. 10, respectively). There were fewer sites involved in recombination events in lineage A than in lineage B (16.9 vs. 67.5%, respectively) (Table 2). The relative effect of mutation and recombination (r/m) was evaluated to 23 (CI_95%_: 23–24) for the *Media* SC (Table 2), but the r/m ratio was of 37 (CI_95%_: 36–40) and of 0.89 (CI_95%_: 0.87–0.90) for lineages B and A, respectively. This showed that recombination is more likely involved in the emergence of clade B than mutation while the two mechanisms of evolution were equally involved in the emergence of clade A.

### Ribosomal multi operon diversity in *Media* SC

The intraclade *rrs* sequence similarity, ranging from 98.7 to 100%, did not exceed the interclade similarity (98.9–100%) and no sequence signature was found (Supplementary Taxonomic Sheet; and Supplementary Table 2). This supported that *rrs* sequencing within *Media* SC lacked of discrimination power. The SLP tree based on almost complete 16S rRNA gene (*rrs*) sequences (1,322 bp) confirmed that ribosomal phylogeny did not robustly discriminate strains from the 3 *Media* SC MLP clades, as generally observed for other closely related species in the genus *Aeromonas* (Supplementary Figure 1). In addition, major incongruences were observed between HK MLP and *rrs* phylogeny (Figure 1 and Supplementary Figure 1).

Independently to the clade, 7 to 11 copies of *rrs* were found in the *Media* SC strains (Figure 1), and this high number of copies represents a potential diversity marker. Chromatogram analysis of the 16S rRNA gene sequences revealed that there were microheterogeneities between *rrs* copies in a single strain (Supplementary Table 2). Sequence microheterogeneities were located within the V3 (457 to 464, and 469 to 476 position in *rrs*) and V6 (1,009 to 1,011, and 1,018 to 1,019 position in *rrs*) regions.

At the *Media* SC level, 27 different PCR-TTGE patterns revealing 14 different V3 sequences in *rrs* (1 to 5 distinct sequences per strain) were detected, and pattern diversity was more frequent in clade A (Supplementary Table 3). TTGE band 15 was present in all strains belonging to clades B and C while observed only for 3 strains belonging to clade A (*P*-value < 0.0001). TTGE band 21 was present in all but two strains belonging to clade A while present in 3 out of the 16 strains belonging to clade B and absent from clade C strains (*P*-value < 0.0001) (Figure 1; Supplementary Table 3).

The *rrs* distribution along the chromosome also varied with 33 different I-*Ceu*I PFGE patterns observed in the studied population. A 400-kb I-*Ceu*I fragment was more frequently observed for strains belonging to clades A and C (74% and 100%, respectively) than for those of clade B (7%) (Figure 1; *P*-value = 0.0002). Clustering obtained according to *rrn* distribution was congruent with MLP clades (Adjusted Wallace Coefficient = 1.00, CI_95%_: 1.00-1.00), while congruence was lower between V3 PCR-TTGE profiles and MLP clades (Adjusted Wallace Coefficient = 0.64, CI_95%_: 0.26–1.00).

Altogether, strains belonging to clades A, B, and C were distinguished by the number and distribution of *rrs* in the chromosome combined or not with V3 heterogeneity. Even if *rrs* sequences were weak phylogenetic markers, ribosomal skeleton and *rrs* copy repertoire were traits related to clade emergence in *Media* SC.

### Comparative genomics in *Media* SC

The genomes from 14 *Media* SC strains (5, 7, and 2 strains affiliated to the clade A, B and C, respectively) were compared. For strains belonging to the same clade, the genomic metrics ANI and *is*DDH values were ≥95.3% and ≥65.1%, respectively, while values ≤ 94.6% and ≤ 61.2% were observed between clades, respectively (Table 3). According to the current rules in taxonomy, the genome metrics affiliated the 3 clades to 3 genomospecies, two of them being already described as taxonomic species, *A. media* and *A. rivipollensis*. The clade/genomospecies “C” was proposed herein as *Aeromonas* sp. genomospecies *paramedia* awaiting further studies for taxonomic species description.

**Table 3:** **Average Nucleotide Identity (ANI) and *in silico* DNA-DNA Hybridization (*is*DDH) for strains belonging to the *Media* complex**.

**Clade**	**Strain**	**G + C content**	**A**					**B**							**C**	
			**1**	**2**	**3**	**4**	**5**	**6**	**7**	**8**	**9**	**10**	**11**	**12**	**13**	**14**
**A**	1- *A. caviae* LMG 13459	61.7%		79.2	73.2	77.4	68.0	53.7	56.6	54.3	53.6	54.1	52.9	52.9	60.6	60.1
	2- BVH40	61.4%	**97.2**		74.1	78.4	68.2	54.1	56.9	54.6	54.2	54.6	53.0	53.0	61.2	61.1
	3- AK202	61.3%	**96.4**	**96.5**		73.6	65.1	57.4	60.5	57.8	57.0	57.5	56.5	56.5	60.0	60.5
	4- *A. media* 76C	61.3%	**97.0**	**97.1**	**96.5**		67.5	54.5	57.2	55.2	54.2	54.9	53.3	53.3	60.5	60.2
	5- *A. hydrophila* 4AK4	62.0%	**95.8**	**95.8**	**95.3**	**95.7**		53.7	55.3	53.9	53.2	54.0	53.3	53.3	61.0	60.6
**B**	6- *A. caviae* LMG 13464	61.3%	**93.2**	**93.2**	**93.9**	**93.3**	**93.1**		74.9	78.9	80.3	79.6	78.8	78.7	57.2	57.0
	7- AK211	61.1%	**93.7**	**93.8**	**94.5**	**93.9**	**93.5**	**96.7**		74.9	74.9	76.7	74.7	74.6	57.9	57.5
	8- *A. media* CECT 4232^*T*^	61.1%	**93.2**	**93.3**	**93.9**	**93.4**	**93.1**	**97.2**	**96.6**		77.9	83.8	79.2	79.2	57.5	57.0
	9- *Aeromonas sp*. CECT 7111	61.6%	**93.1**	**93.2**	**93.8**	**93.2**	**93.1**	**97.4**	**96.7**	**97.1**		78.6	78.5	78.5	57.0	56.8
	10- *A. media* WS	60.7%	**93.2**	**93.2**	**94.0**	**93.3**	**93.2**	**97.2**	**96.8**	**97.7**	**97.2**		79.5	79.4	57.4	57.2
	11– *A. media* ARB13	61.0%	**92.9**	**93.0**	**93.7**	**93.0**	**93.1**	**97.2**	**96.7**	**97.2**	**97.2**	**97.1**		100.0	57.4	56.8
	12– *A. media* ARB20	61.0%	**93.0**	**93.0**	**93.7**	**93.0**	**93.1**	**97.2**	**96.7**	**97.2**	**97.2**	**97.1**	**100.0**		57.3	56.7
**C**	13- 1086C	62.2%	**94.5**	**94.6**	**94.3**	**94.4**	**94.6**	**93.9**	**94.0**	**93.9**	**93.8**	**93.9**	**93.9**	**93.9**		81.9
	14- *A. eucrenophila* UTS 15	61.8%	**94.4**	**94.6**	**94.4**	**94.4**	**94.5**	**93.8**	**93.9**	**93.8**	**93.8**	**93.8**	**93.7**	**93.8**	**97.5**	

*ANI values are displayed in bold in the lower triangle and isDDH values in the upper triangle. The isDDH values displayed are the upper limits of the 95% confidence intervals. Guanine + cytosine content is indicated for each genome (G + C content %)*.

**Figure 3:**
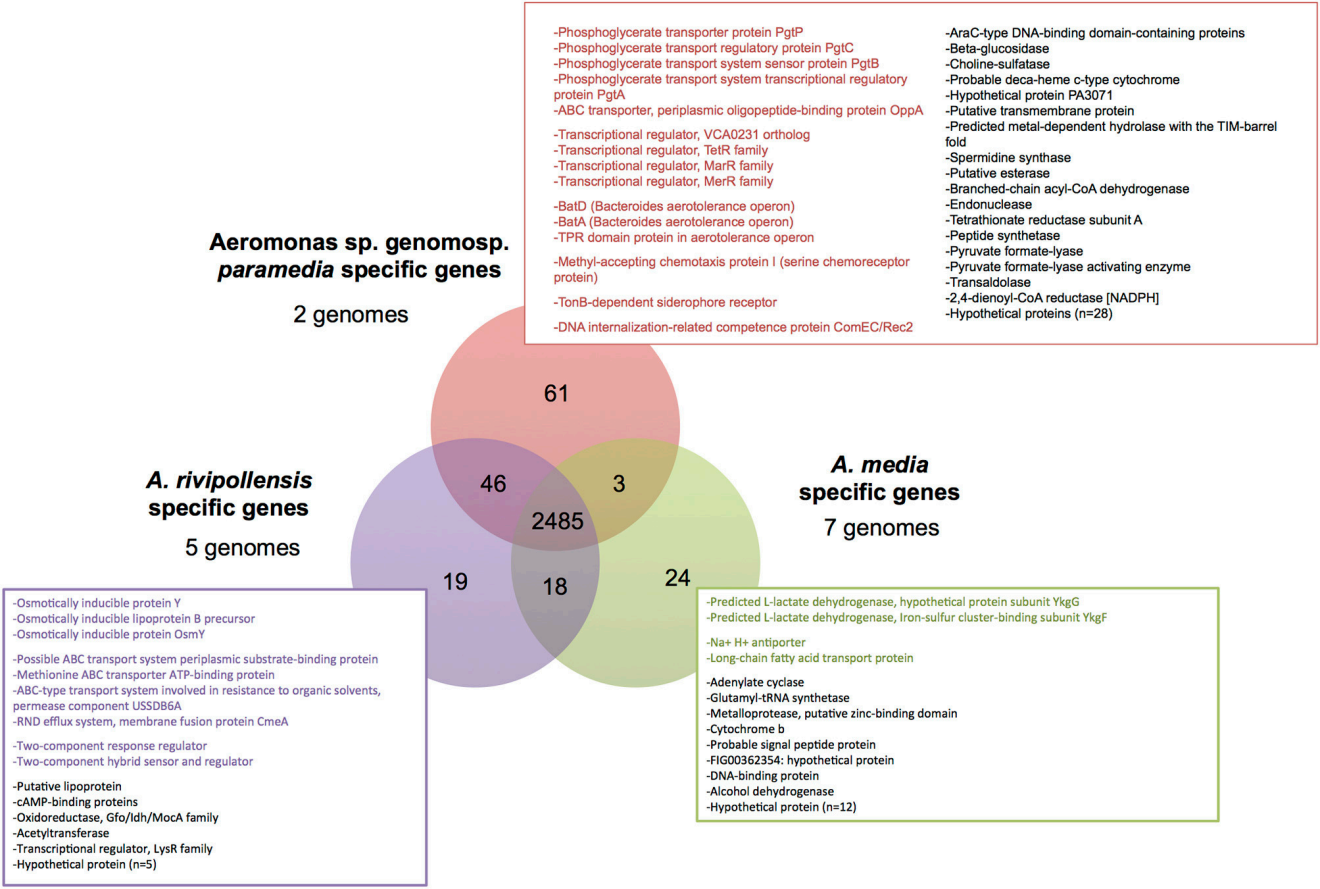
**Venn diagram representing the core-genome of *Media* species complex and lineage conserved core-genome**. The conserved specific genes are indicated for each lineages, and genes likely associated to environmental adaptation and/or change are colored.

The pangenome of *Media* SC was estimated in 7,867 genes, the core-genome in 2,485 genes, the softcore-genome in 3,026 genes, the shell-genome in 1,395 and the cloud-genome in 3,446 genes. The clade-specific genome was analyzed within *Media* SC. It corresponded to the core-genome of each clade that was not shared with any of the two other clades (Figure 3). Nineteen genes were specific to *A. rivipollensis* genomes (Figure 3) especially genes involved in environmental adaptation, including osmotically inducible proteins (*n* = 3), transport systems (*n* = 4) and two-component system proteins (*n* = 2). However, the sole phenotype related to osmoregulation tested herein, the tolerance to NaCl, did not differ between *A. media* and *A. rivipollensis* (Supplementary Taxonomic Sheet). The *A. media* clade-specific genome was composed of 24 genes (Figure 3), among which two subunits of a predicted L-lactate dehydrogenase, two proteins of transport systems and a majority of CDS (Coding DNA Sequences) with unknown function. Phenotypically, the ability to utilize DL-lactate also differentiated *A. media* (clade B) (Figure 1). The specific genome of the genomospecies *paramedia* (clade C) was larger but this was probably due to the comparison of only 2 clade C genomes and should be confirmed on a larger panel of genomes. About half genes (*n* = 29) were annotated as hypothetical proteins in this clade. Among the 32 other genes, half were involved in adaptation to environment or to environment changes: transporters (*n* = 5), transcriptional regulators (*n* = 4), aerotolerance (*n* = 3), chemotaxis (*n* = 1), iron uptake (*n* = 1), and competence (*n* = 1).

Finally, the core-genome of *Media* SC represented 30% of its pan-genome in our study. The clade specific-genomes contained both housekeeping and adaptive genes suggesting that speciation within *Media* SC could be driven by positive selection related to environment conditions.

### *In silico* delineation of habitats and lifestyles within *Media* SC

Considering previous genomic data, habitat and lifestyle within *Media* SC could be questioned, mainly for *A. media* and *A. rivipollensis*. Among 113 *gyrB* sequences retrieved from databases, 85 included the region that we amplified from our collection (positions 298–644). In this region, three specific positions (508, 609, and 627) unambiguously differentiated *A. rivipollensis, A. media* and *Aeromonas* sp. genomospecies *paramedia* and they were used to likely assign the 85 aligned sequences to a clade: 54 were assigned to *A. rivipollensis* and 29 to *A. media*. Together with the strains studied herein (Table 1), the two species were equally recovered from a majority of environmental sources, mostly corresponding to wastewater (29 vs. 34%, *P*-value = 0.7), and soil and river sediments (3 vs. 4%, *P*-value = 0.6). Considering animal sources, we did not observe any difference in the type of host between the species *A. rivipollensis* and *A. media* although data are obviously limited by a low power: fishes (24 vs. 13%, *P*-value = 0.2) and other aquatic inhabitants, e.g., copepods, mussels, crabs and oysters (7 vs. 2%, *P*-value = 0.4), snails (both 4%) and goats (1 vs. 2%, *P*-value = 1.0). The exceptions were strains isolated from pigs that were affiliated to *A. rivipollensis* (16%) only. In addition, there was a trend for a higher prevalence of *A. rivipollensis* in marine or estuarine habitat than the one of *A. media* (15 vs. 6%, *P*-value = 0.076).

The human isolates of *A. rivipollensis* and *A. media* were both found in disease context (both 2%) or during an asymptomatic carriage (2 vs. 4%, *P*-value = 0.6) (Table 1). Despite similar habitats, *A. rivipollensis* strains were more frequently associated with animal or human hosts (44/75) compared with *A. media* strains (13/47) (*P*-value = 0.002).

## Discussion

### Evolution mode in aeromonads and *Media* SC

Bacterial evolution is a major criterion for taxonomy because 16S rRNA (*rrs*) gene-based phylotaxonomy has become since 3 decades a major standard for species delineation and description. Alternative markers are increasingly used when 16S rRNA gene is not enough informative for robust phylogeny. This is particularly the case for bacteria organized in species complex such as *Ralstonia* (Li et al., 2016), *Burkholderia cepacia* (Vandamme and Dawyndt, 2011), *Acinetobacter baumanii* (Cosgaya et al., 2016). Alternative markers are genes encoding housekeeping proteins frequently associated in MLP studies (Roger et al., 2012b) that also allow population structure studies. The use of MLP is recommended in aeromonads taxonomy because 16S rRNA gene is considered of low value for classification of closely related *Aeromonas* spp. in the genus (Martinez-Murcia et al., 1992, 2011). Reasons are both the low *rrs* diversity among related species and the heterogeneity among *rrs* copies in a single species that may surpass the interspecies sequence divergence (Alperi et al., 2008; Roger et al., 2012b; Lorén et al., 2014). Finally, new recommended taxonomy approaches proposed by Chun and Rainey (2014) that consist in analyzing complete genomes with a polyphasic approach, have been applied to *Media* SC.

For the *Media* SC, the use of the sequences of 16 concatenated genes improved the robustness of the phylogeny compared to 7-gene based tree and single gene based trees (Roger et al., 2012a). This confirms that large amount of genetic information may be needed to robustly detect a phylogenetic signal in the genus (Colston et al., 2014), particularly when species are closely related. The use of 16 housekeeping genes gives ML trees congruent with core genome-based phylogeny (Colston et al., 2014) confirming that 16 gene-based MLP is an efficient tool to delineate clades among aeromonads. Moreover, 16 gene-based MLP was congruent with genome metrics determination (ANI and *is*DDH). For the genus *Aeromonas* a good agreement between MLP and DDH (Figueras et al., 2011; Martinez-Murcia et al., 2011) and between MLP, *is*DDH, and ANI (Colston et al., 2014) have been reported. Comparison of MLP tree structure and genome metrics thresholds gives the status of taxonomic species to clades and lineages. In this study, it supported the delineation of *A. rivipollensis* and *A. media* as independent species but also conferred the status of genomospecies to clade C. A temporary non-validated denomination is proposed for the third genomospecies, *Aeromonas* sp. genomospecies *paramedia*, in order stimulate comparison with members of this group in future taxonomic descriptions or reappraisals. *Aeromonas* sp. genomospecies *paramedia* should not be considered as a new species on the basis of the data provided herein and larger number of strains is awaited for a robust species description.

A previous study of temporal diversification suggested that *Aeromonas* has begun to diverge by mutation about 250 My ago and exhibited constant divergence and speciation rates through time and clades (Lorén et al., 2014). This divergence time is considered to be rather slow compared to *E. coli*/*Salmonella* spp. divergence (120 My). One hypothesis to explain slow speciation rates is that recombination events among members of species complexes could blur vertical lineage emergences. Recently, Huddleston et al. (2013) have established that natural transformation could be a mechanism for HGT between environmental *Aeromonas* strains, and have shown that the transformation groups roughly corresponded to phylogroups. In *A. media* SC, MLP showed that mutation signals were blurred by neither homoplasy nor HGT, but that genetic exchanges were linked to clade with higher frequency of recombination inside the clades of *Media* SC. However, it is noteworthy that *A. media* and *A. rivipollensis* differed clearly by their r/m. The emergence of the *A. media* lineage is more influenced by recombination events than *A. rivipollensis*. The speciation in bacterial population could be influenced by differing modes of evolution involved in lineage emergence (Vos and Didelot, 2009), as observed for *A. media* and *A. rivipollensis*. Given their importance in the speciation process (Lassalle et al., 2015), difference in evolution mode between two closely related bacterial populations could be used as a complementary character for new species delineation in an approach of integrative taxonomy (Teyssier et al., 2003; Alauzet et al., 2014).

Despite the development of alternative markers and phylogenomics, ribosomal operons (*rrn*) containing 16S rRNA gene (*rrs*) remain major landmarks of chromosomal structure and bacterial evolution. Indeed, conservation of *rrn* sequence and copy number that protect protein synthesis from major variations appears as a quasi-general rule in the bacterial world. The meaning of the high number and heterogeneity of *rrs* copies in aeromonads remains unknown but there is no doubt that such a diversity in *rrn* content should be linked to particular evolution processes, such as genome dynamics (Teyssier et al., 2003). In *E. coli* mutants, the *rrn* copy number has been experimentally related to living capacities in diverse conditions (Gyorfy et al., 2015). Differences in sequence between copies are less studied but it has been proposed for vibrios and aeromonads (Jensen et al., 2009; Roger et al., 2012a) that sequence variations among *rrn* repertory may achieve fine-tuning of the ribosome function. This could maintain functional diversity and thereby could optimize bacterial adaptation in unstable conditions. RIMOD analysis suggested there was either diversification from ancestral conserved type 21 *rrs* sequence in *A. rivipollensis* and type 15 in *A. media* or, contrarily, differential homogenization of copies by concerted evolution in each species (Liao, 2000; Teyssier et al., 2003). The latter hypothesis fits with the adaptation to more stable ecological conditions and therefore with a speciation process in adequacy to the ecotype concept of species. Facing the lack of diversity of *rrs* bulk sequences (mixing sequences of the different copies) in aeromonads, the combined diversity in *rrs* copy number and sequence, so-called RIMOD, is an interesting marker (Roger et al., 2012b). Since RIMOD, MLP, and ANI showed congruent grouping of strains, an involvement of *rrn* dynamics in *A. media* speciation could be suggested. Finally, our results underline that 16S rRNA genes deserve to be studied in aeromonads taxonomy not by phylogeny approach based on bulk sequences but rather by approaches such as RIMOD that takes into account the diversity in number and in sequence of every copy.

Insights about speciation processes in a population can also be provided by MLP and genomics. The chaperone protein DnaJ participates actively to the response to hyperosmotic and/or heat shocks in a DnaK-independent pathway (Caplan et al., 1993). Among the *Media* SC population (41 isolates), a 3-amino acid deletion in the sequence of protein DnaJ was detected in 95% (*n* = 20) of the strains belonging to *A. rivipollensis* and in 17% (*n* = 3) to *A. media*, while absent from all the strains belonging to other species of the genus. Genomics data from 14 genomes showed that 3 genes presumed to be associated to the osmotic stress response were specific to *A. rivipollensis* genome that potentially acquired them by HGT from *Betaproteobacteria*. Although the absence of homologs does not mean that the function is absent in *A. media*, one scenario suggested by these results is that the type of response to hyperosmotic stress in particular niches could be involved in the speciation of *A. rivipollensis*. We cannot exclude that similar role of positive selection by environmental conditions could be suspected from the annotation of the specific genome of each *Media* SC clades. In fact, most CDS are annotated as “hypothetical proteins” or as protein involved in adaptation to environment and environment changes such as transporters or transcriptional regulators, but this assessment requires further studies.

With the exception of *A. salmonicida* subsp. *salmonicida* that is considered as a specialized fish pathogen (Dallaire-Dufresne et al., 2014) and that present genomic characteristics associated with host adaptation such as gene decay (Reith et al., 2008), habitat and lifestyle have been hardly related to population structure and genomics in the genus *Aeromonas* (Roger et al., 2012a). Overall, the speciation by emergence of genomically differentiated populations without specialized lifestyle, as observed in this study, argues for a versatile behavior. Speciation within the *Media* SC might be mainly driven by general environmental changes such as chemical and physical conditions rather than by adaptation to a particular host. In this study, water and sediments likely appear as primary habitats whereas association with aquatic organisms and vertebrate gut could be an opportunistic lifestyle, sometimes involved in infectious processes.

### Toward integrative and population-based delineation of bacterial species

We proposed herein a comprehensive population study of bacteria affiliated to the species *A. media* (Allen et al., 1983) and to *A. rivipollensis* (Marti and Balcázar, 2015), two species that were initially defined on the basis of 15 *A. media* strains isolated around the same trout farm and 2 *A. rivipollensis* isolated in the same Spanish river. Several characteristics determined at the time of the species description have been described more precisely herein on a larger panel of strains from diverse habitats. *A. media* should be now considered as motile and *A. media* and *A. rivipollensis* as rare brown pigment producers. The motility and production of pigment were the only phenotypic characters used to differentiate *A. rivipollensis* from *A. media* by Marti and Balcázar (2015) when pigment production is only a typical reaction of the type strain of *A. media* but not of the other strains. The recent description of *A. rivipollensis* is emblematic of the taxonomic pitfalls of considering a limited number of isolates and of considering strains of the closely recognized species limited to the sole type strain for novel species characterization. Therefore, an emendation of *A. media and A. rivipollensis* description is necessary and proposed below.

This argues for further strengthening the taxonomic recommendations toward a population-based taxonomy. The number of strains included in a taxonomic description is not a sufficient criterion for robust species description; the representativeness of the population should also be taken into account, as advised by Christensen et al. (2001) and as detailed in the Report of the *ad hoc* committee for the re-evaluation of the species definition in bacteriology (Stackebrandt et al., 2002). Although representativeness may sometimes be complex to achieve because it is difficult to know the population and its habitats at the stage of species description, redundancy should be strictly fought and diversity actively sought. The strains studied herein covered the known lifestyle diversity of strains affiliated to *Media* SC as confirmed by databank survey.

Polyphasic approach is recommended since a long time for bacterial taxonomy (e.g., Colwell, 1970; Figueras et al., 2011). However, the current bacterial taxonomy remains mainly numerical and phenetic, even if the reconstruction of a phylogenetic tree containing the new taxon is generally required for species description. The phylogenetic tree is mainly used to easily visualize the relationships between the new taxon and known taxa rather than for true evolutionary analyses. Multi-locus genetics and genomics generate large amount of data that should be used for a more integrative purpose, i.e., integration of population biology and evolutionary disciplines into taxonomy (Padial et al., 2010), in order to circumscribe a population that gains the rank of species in its biological and evolutionary dimensions in addition to the barcoding that defines its taxonomic frame. Therefore, integrative taxonomy will provide advances for the definition of species or other significant units in the domain *Bacteria*, particularly by providing data about genomic (Chun and Rainey, 2014) and/or ecological speciation (Lassalle et al., 2015) beside numerical definition of taxa. This is particularly true for genera in which the species are closely related or organized in complexes of species, i.e., groups of species with unclear boundaries as observed for *Aeromonas, Vibrio, Acinetobacter, Pseudomonas, Burkholderia*, and several enterobacterial genera.

### Emended description of *Aeromonas rivipollensis*, Marti and Balcazar 2016

The description is as done by Marti and Balcázar (2015) with the exceptions that strains are able to growth at 4°C, and the growth is variable at 3% NaCl but not at 5% NaCl. Strains do not use citrate. Hydrolysis of gelatin is variable. Acid is produced by fermentation from L-arabinose. Acid production from lactose or amygdalin is variable.

In addition, strains are motile but do not swarm. No hemolysis is observed on sheep blood agar at 35°C. Acid is produced by fermentation from D-mannose, D-cellobiose. The strains are negative for production of butanediol, DL-lactate and gas from glucose. Strains are susceptible to cefoxitin.

The key phenotypic characters that differentiate the species from the close relative *A. media* are the absence of DL-lactate utilization and cefoxitin susceptibility. For routine identification purpose, when genomics data are unavailable, *gyrB* combined with *radA* sequencing is efficient to separate *A. rivipollensis* from *A. media* and from others genomospecies in the genus.

### Emended description of *Aeromonas media* Allen et al. 1983

The description is as done by Allen et al. (1983) with the exceptions that swimming motility is observed and that diffusible brown, non-fluorescent pigment produced by the type strain is rarely produced by other strains. In addition, growth occurs variably in 3% (wt./vol.) sodium chloride, variable response is observed for arginine dihydrolase, and strains utilize sucrose as sole carbon sources for energy and growth. Strains are resistant to cefoxitin.

The species can be differentiated from close relatives by the DL-lactate utilization and resistance to cefoxitin. For routine identification purpose, when genomic data are unavailable, *gyrB* combined with *radA* sequencing is efficient to separate *A. media* from *A. rivipollensis* and from others genomospecies in the genus. Compared to *A. rivipollensis, A. media* harbors a specific combination of three genes presumed to be associated to DL-lactate utilization (L-lactate permease, L-lactate dehydrogenase and D-lactate dehydrogenase).

The DNA G+C content of the type strain, determined from the genome sequences, was 61.3%, close to the rate of the original description obtained by denaturation experiments, which was 62.3 ± 0.2%.

## Author contributions

Conceived and designed the study: BL, HM, and EJ; Designed and performed the acquisition of clinical isolate and environmental collection: BL, MF, and FP; Performed the microbial analyses: ET, JK, FR, and FL; Performed acquisition and analyses of whole genome data: SC, ET, and JG; Analyzed and interpreted the data: ET, FR, JK, HM, EJ, and BL (microbial data), ET, SC (WGS); Discussed the taxonomical considerations: ET, BL, MF, HM, and EJ; Drafted the paper: ET, EJ, and BL; Critically revised the manuscript: HM, JG, MF, SC, FP, and FR. All authors read and approved the final manuscript.

### Conflict of interest statement

The authors declare that the research was conducted in the absence of any commercial or financial relationships that could be construed as a potential conflict of interest.
