# Supplementary material for: Delineation of Taxonomic Species within Complex of Species: Aeromonas media and Related Species as a Test Case
Source: Front Microbiol. 2017 Apr 18;8:621. doi: 10.3389/fmicb.2017.00621 (PMC5394120; doi:10.3389/fmicb.2017.00621)
Supplement: Supplementary file 1 [file Table1.DOCX]

**Supplementary Table 1. General characteristics of the 14 *Aeromonas* genomes used in the study**

| Strain | Clade in MLP | Genome size (Mbp) | No of scaffolds | Average genome coverage | N50 (nt.)† | G+C content (%) | No of predicted CDS | Level of assembly | Accession number | Reference |
| --- | --- | --- | --- | --- | --- | --- | --- | --- | --- | --- |
| *A. caviae* LMG 13459 | A | 4.49 | 111 | 76 | 107760 | 61.7% | 4,091 | IHQ | PRJEB12346 | This study |
| *A. media* BVH40 | A | 4.70 | 123 | 79 | 105841 | 61.4% | 4,204 | IHQ | PRJEB9017 | Mosser et al., 2015 |
| AK202 | A | 4.66 | 118 | 68 | 89731 | 61.3% | 4,230 | IHQ | PRJEB12343 | This study |
| *A. media* 76C | A | 4.69 | 137 | 79 | 93768 | 61.3% | 4,255 | IHQ | PRJEB8966 | Mosser et al., 2015 |
| *A. hydrophila* 4AK4 | A | 4.53 | 1 | 744 | 4,527,993 | 62.0% | 4,065 | complete | CP006579.1 | Gao et al., 2013 |
| *A. caviae* LMG 13464 | B | 4.45 | 99 | 87 | 103746 | 61.3% | 4,014 | IHQ | PRJEB12347 | This study |
| AK211 | B | 4.52 | 150 | 92 | 79,182 | 61.1% | 4,065 | IHQ | PRJEB12344 | This study |
| *A. media* CECT 4232^T^ | B | 4.48 | 233 | 60 | 37,608 | 61.1% | 4,075 | IHQ | PRJEB7032 | Colston et al., 2014 |
| *Aeromonas sp.* CECT 7111 | B | 4.41 | 92 | 70 | 108504 | 61.6% | 3,998 | IHQ | PRJEB12345 | This study |
| *A. media* WS | B | 4.78 | 1 | 210 | 4,777,154 | 60.7% | 4,338 | complete | CP007567.1 | Chai et al., 2012 |
| *A. media* ARB13 | B | 4.61 | 180 | 518 | 65,784 | 61.0% | 4,200 | HQ | PRJNA260228 | Kenzaka et al., 2014 |
| *A. media* ARB20 | B | 4.62 | 185 | 271 | 72,353 | 61.0% | 4,216 | HQ | PRJNA260227 | Kenzaka et al., 2014 |
| 1086C | C | 4.46 | 128 | 99 | 78349 | 62.2% | 4,086 | IHQ | PRJEB12349 | This study |
| *A. eucrenophila* UTS 15 | C | 4.69 | 152 | 142 | 67413 | 61.8% | 4,229 | IHQ | PRJEB12350 | This study |

CDS: Coding DNA Sequence; MLP: Multi-Locus Phylogeny; IHQ: Improved High quality draft genome; HQ: High quality draft genome

†The *N*50 (reported in nucleotides) represents the smallest of the largest contigs covering 50% of the total size of all contigs.
