# Supplementary material for: Delineation of Taxonomic Species within Complex of Species: Aeromonas media and Related Species as a Test Case
Source: Front Microbiol. 2017 Apr 18;8:621. doi: 10.3389/fmicb.2017.00621 (PMC5394120; doi:10.3389/fmicb.2017.00621)
Supplement: Supplementary file 2 [file Table2.DOCX]

**Supplementary Table 2.** **Variable nucleotide positions of the 16S rRNA gene in the 3 clades of the *A. media* species complex and in the other currently recognized *Aeromonas* species (type strains).** Numbers are homologous positions following the numbering system in *E. coli.* Dash indicates the same nucleotide as that of clade A*.* For the *Media* complex, each microheterogeneity observed from 16S rRNA gene chromatograms is reported by a degenerate base symbol (IUPAC nomenclature) and polymorphic positions between strains from the same genomic group are separated by slash.

|  | 154-156 | 250 | 457-464 | 469-476 |
| --- | --- | --- | --- | --- |
| Clade A | AGT | T | TG R/G/A Y/T A/R S/C/G CT | C/M/A G/S/C T/K G/R/A T/C/Y C/T A R/A/G |
| Clade B | - | **-** | - A T R/G C - | C G/S Y/C R/A T C/Y - G |
| Clade C | - | **-** | - A T G C - | C G Y R/A T C - G |
| *A. allosaccharophila* CECT 4199^T^ (S39232) | - | A | - G T A G CG | A C T G C C - G |
| *A. aquatica* CECT 8025^T^ (HG970952) | - | A | - A T G C - | C G C A T C - G |
| *A. australiensis* CECT 8023^T^ (HEG11955) | TAC | A | - G T A G CG | A C T G C C - G |
| *A. bestiarum* CIP 7430^T^ (X60406) | - | A | - G C G C - | C G T G T C - A |
| *A. bivalvium* CECT 71113^T^ (DQ504429) | - | A | - C T G G - | T C C A G C - T |
| *A. cavernicola* CECT 7862^T^ (HQ436040) | TAC | A | - G T A G CG | A C T G C C - G |
| *A. caviae* NCIMB 13016^T^ (X60408) | - | A | CA G T A G - | T C T G C T G G |
| *A. dhakensis* LMG 19562^T^ (AJ508765) | - | A | CA G T A G - | T C T G C T G A |
| *A. diversa* CECT 4254^T^ (GQ365710) | TAC | - | - G T A G CG | A C T G C C - G |
| *A. encheleia* CECT 4342^T^ (HQ832414) | - | A | - A T G C - | C G T A T C - A |
| *A. enteropelogenes* CECT4487^T^ (NR_116026) | - | A | CA G T A G - | T C T G C T G G |
| *A.eucrenophila* NCIMB 74^T^(X60411) | - | A | - A T G C - | C G C A T C - G |
| *A. finlandiensis*  CECT 8028^T^ (LM654283) | TAC | A | GG T T G G - | C C C A A T C G |
| *A. fluvialis* CECT 7401^T^ (FJ230078) | TAC | A | - C T A C CG | A C T G C C - G |
| *A. hydrophila* ATCC 7966^T^ (X60404) | - | A | - A T G C - | C G T A T C - A |
| *A. jandaei* ATCC 49568^T^ (X60413) | TAC | A | CA G T A G - | T C T G C T G G |
| *A. lacus*  CECT 8024^T^ (HG970953) | TAC | A | - G T A G - | T C T G C C - G |
| *A. molluscorum* CECT 5864^T^ (AY532691) | - | A | - A T G C - | C G C A T C - A |
| *A. piscicola* CECT 7443^T^ (HQ832417) | - | A | - G C G C - | C G T G T C - A |
| *A. popoffii* CECT 5176^T^ (HQ832415) | - | A | - T T G G - | C C C A G C - A |
| *A. rivuli* CECT 7518^T^ (FJ976900) | - | A | - G C A G - | T C T G T C - A |
| *A. salmonicida* NCIMB 1102^T^ (X60405) | - | A | - G C G C - | C G T G T C - A |
| *A. sanarellii* CECT 7402^T^ (FJ230076) | - | A | CA G T A G - | C C T G C T G G |
| *A. schubertii* ATCC 43700^T^ (X60416) | TAC | **-** | - G T G G TT | C C T G C C - G |
| *A. simiae* CIP 107798^T^ (GQ860945) | TAC | **-** | - G T A G - | A C T G C C - G |
| *A. sobria* NCIMB 12065^T^ (X60412) | - | A | - G C A G - | T C T G T C - G |
| *A. taiwanensis* CECT 7403^T^ (FJ230077) | - | A | CA G T A G - | T C T G C T G A |
| *A. tecta* CECT 7082^T^ (HQ83241) | TAC | A | - A T G C - | C G C A T C - G |
| *A. veronii* ATCC 35624^T^ (X60414) | TAC | A | - G T A G - | A C T G C C - G |
