# Supplementary material for: Delineation of Taxonomic Species within Complex of Species: Aeromonas media and Related Species as a Test Case
Source: Front Microbiol. 2017 Apr 18;8:621. doi: 10.3389/fmicb.2017.00621 (PMC5394120; doi:10.3389/fmicb.2017.00621)
Supplement: Supplementary file 3 [file Table3.DOCX]

**Supplementary Table 3.** **16S rRNA PCR/TTGE patterns for 37 strains of the *Media* complex.** 26 PCR-TTGE patterns detected 15 different sequences of V3 region of 16S rRNA gene (1 to 5 distinct sequences per strain). Results are presented according to the structure of the population observed with MLP analysis (3 clades). TTGE bands and profiles were numbered according to Roger et al. (2012) with increment for newly described bands (KP717966 to KP718060 and KX553956 to KX553959). TTGE profiles are indicated by a combination of band number separated by a + sign. Bold type indicates sequences 15 and 21 mostly conserved in clades B/C and A, respectively.

| MLP clade | 16S rDNA PCR-TTGE Pattern | Strain |
| --- | --- | --- |
| A | **21** | AK202 |
|  | 11+**21** | AH31, CR4.2-17C |
|  | 11+20 | BVH40 |
|  | 20+**21** | CCM 4242, AK210 |
|  | **21**+41 | R1 |
|  | **21**+46 | 76C |
|  | 11+20+**21** | ADV137a |
|  | 11+**21**+42 | LL6-17C |
|  | **15**+**21**+42 | M2C164 |
|  | 20+**21**+42 | M2C185, BVH17 |
|  | 20+**21**+43 | R100 |
|  | 1+20+**21**+41 | 417-16G |
|  | 11+13+20+**21** | LMG 26313^T^ |
|  | 11+20+**21**+42 | M2C205 |
|  | 20+**21**+42+44 | AK208 |
|  | 2+**15**+20+**21**+30 | SS1-15D |
|  | **15**+20+**21**+30+45 | LMG 13459 |
| B | **15** | CECT 4232^T^, CECT 4234, CR4-18pb |
|  | **15**+20 | RT11-17Ga |
|  | **15**+42 | D84402, T41-7, LMG 13464 |
|  | 2+**15**+20 | RT10-17Ga |
|  | **15**+20+**21** | SEL1-18A, LL4.4-18D |
|  | **15**+20+42 | T41-3, BVH83 |
|  | **15**+20+47 | AK207, AK211 |
|  | 2+**15**+20+42 | CECT 7111 |
| C | **15**+42 | UTS 15 |
|  | **15**+30+42+44 | 1086C |

Roger, F., Lamy, B., Jumas-Bilak, E., Kodjo, A., colBVH study group, and Marchandin, H. (2012). Ribosomal multi-operon diversity: an original perspective on the genus Aeromonas. *PloS One* 7, e46268. doi:10.1371/journal.pone.0046268.
