# Supplementary material for: Delineation of Taxonomic Species within Complex of Species: Aeromonas media and Related Species as a Test Case
Source: Front Microbiol. 2017 Apr 18;8:621. doi: 10.3389/fmicb.2017.00621 (PMC5394120; doi:10.3389/fmicb.2017.00621)
Supplement: Supplementary file 4 [file DataSheet1.DOCX]

SUPPLEMENTARY TAXONOMIC SHEET

Phenotypic data useful for species emendation are described in this taxonomic sheet. Common traits among *A. media*, *A. rivipollensis* and the genomospecies *paramedia* are also provided as well as sequence identity level among taxa.

**Intraclade, interclade and interspecies gene sequence similarities**

Table 1 and 2 of the supplementary taxonomic sheet provide intraclade, interclade and interspecies gene sequence similarities (%).

Note that the multi-locus phylogenetic analysis of the concatenated sequences of *gyrB* and *radA* genes showed a more robust delineation of the clades (bootstraps ≥ 88%), despite the intraclade and interclade similarity overlapped for the concatemer from these two genes (≥ 96.2% and ≤ 97.5%, respectively; Table 2 of the supplementary taxonomic sheet).

Methods for phenotypic characterization

Metabolic tests performed at 35 +/- 2 °C on *A. media* and *A. rivipollensis* isolates were those included in API 20E strips (BioMérieux, France), gas production from glucose (BBL Kligler iron agar slants, Beckton-Dickinson, USA) and DL-lactate utilization (Altwegg et al., 1990). Additional metabolic characterization of selected strains included carbohydrate fermentation (API 50 CH strips, BioMérieux, France), pyrazinamidase activity, ammonium chloride utilization, and acid production from D-mannose and D-lactose (MacFaddin, 1993). NaCl Minimum Inhibitory Concentration (MIC) using microbroth dilution method was determined as described elsewhere (Michon et al., 2014). Pigment production was observed on Trypticase Soy Agar, and hemolysis on sheep blood agar, after incubation for 48 h at 35 °C. To assess motility, overnight cultures on Trypticase Soy Broth at 35 °C under agitation at 150 rpm were inspected by light microscopy, adjusted to 0.1 OD_600nm_ and 5μL were spotted at the center of swarm agar (Nutrient broth Difco^TM^, USA; D-glucose 0.5%; agar 0.5%) and swim agar (LB Broth Lennox Difco^TM^, agar 0.3%) plates. Plates were incubated face up at 30 °C for 4 days and the motility ring diameters were measured daily. Antibiotyping was performed using the disk diffusion method on Mueller-Hinton agar according to the 2013 guidelines of the Antibiogram Committee of the French Society for Microbiology (CA-SFM) and *Enterobacteriaceae* breakpoints.

**Phenotypic characters of *A. media*, *A. rivipollensis* and *Aeromonas* sp. genomospecies *paramedia***

Despite similar phenotypes (Table 4 of the supplementary taxonomic sheet), strains from the two main genomospecies were discriminated by their ability to utilize DL-lactate. This result was in agreement with the presence of L-lactate permease, L-lactate dehydrogenase and D-lactate dehydrogenase in the genomes of clade B strains only. All the antimicrobial susceptibility profiles were similar, with the exception of cefoxitin susceptibility observed for all the tested members of clade A (n=20) and for 2 out of the 15 strains tested from clade B (*P* value < 0.0001; Table 3 of the supplementary taxonomic sheet).

**Common traits in *A. media*, *A. rivipollensis* and *Aeromonas* sp. genomospecies *paramedia***

The estimated size of *A. media* genomes ranged from 4.41Mb (*Aeromonas* sp. CECT 7111) to 4.78Mb (*A. media* WS). The DNA G+C mol% of the genomes ranged from 60.7% to 62.2% without differences between clades (Table 3). The three clades displayed an overall high metabolic homogeneity in the API20E and API50CH. They have all showed acid production from D-mannitol and D-saccharose. Varying characters were strain-dependent within each clade (Table 4 of the supplementary taxonomic sheet). The susceptibility of the 36 strains tested to 10 antibiotics (amoxicillin, co-amoxiclav, ticarcillin, ticarcillin plus clavulanic acid, cefalotin, cefotaxime, imipenem, ciprofloxacin, gentamicin and cotrimoxazole) was similar between clades (Table 3 of the supplementary taxonomic sheet). NaCl values ranged from 3 to 5%, with no difference between clades (*P* value=0.5). Hemolysis on sheep blood agar was negative for all the tested strains (n=37) and only three of the isolates (*A. media* CECT 4232^T^, *A. media* CECT 4234 and 76C) produced a brown diffusible pigment on TSA. All the strains tested were motile with the exception of the strain SEL1-18A (clade B). Swimming properties, fully developed after 96 h, were highlighted for all motile strains with the exception of three and one strains in clades B and C, respectively. None of the isolates did swarm.

**Table 1 of the supplementary taxonomic sheet.** Intraclade, interclade and interspecies 16S rRNA gene sequence similarities (%) of strains affiliated to *Media* species complex with other clades and other type strains of currently recognized *Aeromonas* species. In bold, the most similar *Aeromonas* species out of the complex *Media*.

|  | Clade A, %  n=21 | Clade B, %  n=16 | Clade C, %  n=2 |
| --- | --- | --- | --- |
| Clade A (n=21) | 98.7-100.0 | 98.9-100.0 | 99.0-99.7 |
| Clade B (n=16) | 98.9-100.0 | 99.0-100.0 | 99.1-100.0 |
| Clade C (n=2) | 99.0-99.7 | 99.1-100.0 | 99.6 |
| *A. allosaccharophila* CECT 4199^T^ (S39232) | 98.2-99.0 | 98.3-98.9 | 98.3-98.4 |
| *A. aquatica* CECT 8025^T^ (HG970952) | 98.2-98.9 | 98.5-99.2 | 99.0 |
| *A. australiensis* CECT 8023^T^ (HEG11955) | 97.8-98.5 | 97.8-98.4 | 97.8-97.9 |
| *A. bestiarum* CIP 7430^T^ (X60406) | 98.1-98.8 | 98.4-98.7 | 98.5-98.6 |
| *A. bivalvium* CECT 71113^T^ (DQ504429) | 98.1-98.6 | 98.3-98.6 | 98.3-98.4 |
| *A. cavernicola* CECT 7862^T^ (HQ436040) | 97.2-98.1 | 97.2-97.8 | 97.2-97.3 |
| *A. caviae* NCIMB 13016^T^ (X60408) | 98.5-99.3 | 98.7-99.3 | 98.7 |
| *A. dhakensis* LMG 19562^T^ (AJ508765) | 98.5-99.3 | 98.6-99.3 | 98.6-98.7 |
| *A. diversa* CECT 4254^T^ (GQ365710) | 97.4-98.2 | 97.5-98.2 | 97.5-97.6 |
| *A. encheleia* CECT 4342^T^ (HQ832414) | 98.4-99.0 | 98.5-99.0 | 98.7-98.9 |
| *A. enteropelogenes* CECT4487^T^ (NR_116026) | 98.4-99.1 | 98.6-99.1 | 98.6-98.7 |
| *A. eucrenophila* NCIMB 74^T^ (X60411) | 98.4-99.0 | 98.4-99.1 | 98.8-99.0 |
| *A. finlandiensis*  CECT 8028^T^ (LM654283) | 97.6-98.1 | 97.9-98.2 | 97.9-98.1 |
| *A. fluvialis* CECT 7401^T^ (FJ230078) | 97.8-98.4 | 97.9-98.4 | 97.9-98.0 |
| ***A. hydrophila* ATCC 7966^T^ (X60404)** | **99.0-99.6** | **99.3-99.7** | **99.6** |
| *A. jandaei* ATCC 49568^T^ (X60413) | 97.7-98.4 | 97.8-98.4 | 97.8-97.9 |
| *A. lacus*  CECT 8024^T^ (HG970953) | 97.7-98.4 | 97.8-98.3 | 97.8 |
| *A. molluscorum* CECT 5864^T^ (AY532691) | 98.3-98.7 | 98.5-99.0 | 98.8 |
| *A. piscicola* CECT 7443^T^ (HQ832417) | 98.1-98.8 | 98.4-98.7 | 98.5-98.6 |
| *A. popoffii* CECT 5176^T^ (HQ832415) | 98.2-98.7 | 98.5-98.8 | 98.6 |
| *A. rivuli* CECT 7518^T^ (FJ976900) | 98.1-98.9 | 98.2-98.7 | 98.2-98.3 |
| *A. salmonicida* NCIMB 1102^T^ (X60405) | 98.1-98.7 | 98.4-98.7 | 98.4-98.5 |
| *A. sanarellii* CECT 7402^T^ (FJ230076) | 98.4-99.1 | 98.6-99.0 | 98.6 |
| *A. schubertii* ATCC 43700^T^ (X60416) | 97.2-98.0 | 97.5-97.8 | 97.5 |
| *A. simiae* CIP 107798^T^ (GQ860945) | 97.1-98.0 | 97.2-97.8 | 97.2 |
| *A. sobria* NCIMB 12065^T^ (X60412) | 98.2-98.8 | 98.3-98.7 | 98.4 |
| *A. taiwanensis* CECT 7403^T^ (FJ230077) | 98.4-99.1 | 98.4-99.1 | 98.4 |
| *A. tecta* CECT 7082^T^ (HQ83241) | 97.9-98.6 | 97.9-98.7 | 98.4-98.5 |
| *A. veronii* ATCC 35624^T^ (X60414) | 98.1-99.0 | 98.1-98.7 | 98.1-98.2 |

**Table 2 of the supplementary taxonomic sheet.** Intraclade, interclade and interspecies housekeeping gene sequence similarities (minimum - maximum, %). The discriminant similarity values are written in bold. The interspecies similarity was determined from comparison with the 53 strains included in the study that belonged to the 28 species of *Aeromonas* other than strains affiliated to the complex *Media*.

| Gene |  | Clade A | Clade B | Clade C |
| --- | --- | --- | --- | --- |
| *atpD* | intraclade similarity | 96.0-100.0 | 99.0-100.0 | 99.2 |
|  | interclade similarity | 96.8-99.4 | 96.8-99.4 | 97.0-99.4 |
|  | interspecies similarity | 90.0-99.2 | 90.6-97.6 | 91.4-97.6 |
| *dnaJ* | intraclade similarity | 96.2-100.0 | 95.5-100.0 | 98.4 |
|  | interclade similarity | 94.9-100.0 | 94.9-100.0 | **95.2-97.4** |
|  | interspecies similarity | 80.5-93.5 | **81.0-94.4** | **82.0-93.8** |
| *dnaK* | intraclade similarity | 97.0-100.0 | 90.0-100.0 | 98.8 |
|  | interclade similarity | 90.3-100.0 | 90.3-100.0 | **90.7-98.1** |
|  | interspecies similarity | 90.6-97.0 | 84.8-96.9 | 90.1-96.0 |
| *dnaX* | intraclade similarity | 91.5-100.0 | 94.7-100.0 | 99.3 |
|  | interclade similarity | 90.9-97.7 | 90.9-99.7 | 93.5-99.7 |
|  | interspecies similarity | 87.0-99.5 | 87.9-94.9 | 88.9-95.1 |
| *gltA* | intraclade similarity | 93.0-100.0 | 97.6-100.0 | 98.3 |
|  | interclade similarity | 93.3-99.0 | 93.3-98.3 | 94.4-99.0 |
|  | interspecies similarity | 85.4-97.4 | 86.1-96.9 | 86.3-97.2 |
| *groL* | intraclade similarity | 96.6-100.0 | 97.0-100.0 | 98.8 |
|  | interclade similarity | 95.2-100.0 | 94.9-100.0 | **94.9-97.4** |
|  | interspecies similarity | 84.7-96.8 | 85.6-96.0 | 84.7-95.6 |
| *gyrA* | intraclade similarity | 94.2-100.0 | 98.0-100.0 | 99.7 |
|  | interclade similarity | 94.2-99.7 | 94.2-99.5 | 94.8-99.7 |
|  | interspecies similarity | 89.1-99.7 | 89.1-98.2 | 89.9-98.2 |
| *gyrB* | intraclade similarity | 96.6-99.7 | 96.2-100.0 | 97.7 |
|  | interclade similarity | 94.6-98.0 | 94.6-98.0 | 95.8-97.7 |
|  | interspecies similarity | **86.3-95.3** | **86.3-94.1** | **86.9-93.6** |
| *metG* | intraclade similarity | 97.6-99.8 | 94.6-100.0 | 99.2 |
|  | interclade similarity | 95.2-99.4 | 95.2-99.4 | **96.0-98.6** |
|  | interspecies similarity | **84.9-93.0** | **84.3-93.2** | **85.3-93.6** |
| *ppsA* | intraclade similarity | 92.3-99.0 | 93.2-100.0 | 98.5 |
|  | interclade similarity | 91.4-97.2 | 91.4-97.2 | 92.5-95.7 |
|  | interspecies similarity | 81.0-96.0 | 81.1-94.5 | 81.1-93.6 |
| *radA* | intraclade similarity | 93.0-100.0 | 98.5-100.0 | 99.7 |
|  | interclade similarity | 93.0-99.7 | 93.0-99.7 | 93.0-97.2 |
|  | interspecies similarity | 79.8-93.5 | **79.5-92.8** | **79.8-92.8** |
| *recA* | intraclade similarity | 95.9-100.0 | 95.1-100.0 | 98.8 |
|  | interclade similarity | 94.6-99.6 | 94.6-99.6 | **95.4-97.1** |
|  | interspecies similarity | 87.7-95.9 | 86.9-95.4 | 88.1-95.8 |
| *rpoB* | intraclade similarity | 98.3-100.0 | 97.4-100.0 | 100.0 |
|  | interclade similarity | 96.9-100.0 | 96.9-100.0 | 97.6-100.0 |
|  | interspecies similarity | 88.9-97.8 | 88.7-97.6 | **89.2-96.7** |
| *rpoD* | intraclade similarity | 95.2-100.0 | 96.6-100.0 | 98.8 |
|  | interclade similarity | 94.6-99.8 | 94.6-99.8 | 94.8-99.6 |
|  | interspecies similarity | **78.7-94.0** | **78.3-94.0** | **79.4-93.4** |
| *tsf* | intraclade similarity | 97.8-100.0 | 97.3-100.0 | 99.4 |
|  | interclade similarity | 97.2-100.0 | 97.3-100.0 | **96.7-98.1** |
|  | interspecies similarity | 90.2-97.3 | 90.8-97.6 | **90.8-96.2** |
| *zipA* | intraclade similarity | 70.7-100.0 | 72.3-100.0 | 98.6 |
|  | interclade similarity | 71.4-100.0 | 71.4-99.7 | 71.4-99.5 |
|  | interspecies similarity | 37.5-100.0 | 38.2-100.0 | 38.2-83.7 |
| *gyrB*-*radA* concatemer | intraclade similarity | 96.2-99.3 | 97.3-100.0 | 98.4 |
|  | interclade similarity | 94.4-97.5 | 94.4-97.5 | 95.3-97.2 |
|  | interspecies similarity | **84.1-92.5** | **84.1-91.9** | **84.7-92.1** |

**Table 3 of the supplementary taxonomic sheet.** Antimicrobial susceptibility testing results of the strains affiliated to the complex *Media*. 37 strains were tested except for cefalotin, cefotaxime and gentamicin (n=36). Breakpoints were considered according to the 2013 guidelines of the Antibiogram Committee of the French Society for Microbiology (CA-SFM) and *Enterobacteriaceae* breakpoints. In bold, the susceptibility to cefoxitin that differentiates the clade A from the clade B (*P* value < 0.0001).

| Antimicrobial agent | Disk charge (μg) | ZD upper breakpoint (mm) |  | Clade A  n=20 (%) | |  | Clade B  n=15 (%) | |  | Clade C  n=2 | |
| --- | --- | --- | --- | --- | --- | --- | --- | --- | --- | --- | --- |
|  |  |  |  | S | I+R |  | S | I+R |  | S | I+R |
| Amoxicillin | 25 | ≥ 19 |  | 0 (0) | 20 (100) |  | 2 (13) | 13 (87) |  | 0 | 2 |
| Co-amoxiclav | 20/10 | ≥ 21 |  | 3 (15) | 17 (85) |  | 3 (20) | 12 (80) |  | 0 | 2 |
| Ticarcillin | 75 | ≥ 24 |  | 1 (5) | 19 (95) |  | 3 (20) | 12 (80) |  | 0 | 2 |
| Ticarcillin + clavulanic acid | 75/10 | ≥ 24 |  | 2 (10) | 18 (90) |  | 3 (20) | 12 (80) |  | 0 | 2 |
| Cefalotin | 30 | ≥ 18 |  | 4 (21) | 15 (79) |  | 2 (13) | 13 (87) |  | 0 | 2 |
| **Cefoxitin** | **30** | **≥ 22** |  | **20 (100)** | **0 (0)** |  | **2 (13)** | **13 (87)** |  | **1** | **1** |
| Cefotaxime | 30 | ≥ 26 |  | 18 (95) | 1 (5) |  | 13 (87) | 2 (13) |  | 2 | 0 |
| Imipenem | 10 | ≥ 24 |  | 20 (100) | 0 (0) |  | 14 (93) | 1 (7) |  | 2 | 0 |
| Ciprofloxacin | 5 | ≥ 25 |  | 19 (95) | 1 (5) |  | 13 (87) | 2 (13) |  | 2 | 0 |
| Gentamicin | 15 | ≥ 18 |  | 19 (100) | 0 (0) |  | 15 (100) | 0 (0) |  | 2 | 0 |
| Cotrimoxazole | 1.25/ 23.75 | ≥ 16 |  | 20 (100) | 0 (0) |  | 14 (93) | 1 (7) |  | 2 | 0 |

Abbreviations: ZD, Zone-diameter; S, Susceptible; I, Intermediate; R, Resistant.

**Table 4 of the supplementary taxonomic sheet.** Phenotypic characteristics of *A. rivipollensis*, *A. media* and *Aeromonas* sp. genomospecies *paramedia* and of the other members of the genus.

| Character | **A** | **B** | **C** | 1 | 2 | 3 | 4 | 5 | 6 | 7 | 8 | 9 | 10 | 11 | 12 | 13 | 14 | 15 | 16 | 17 | 18 | 19 | 20 | 21 | 22 | 23 | 24 | 25 | 26 | 27 |
| --- | --- | --- | --- | --- | --- | --- | --- | --- | --- | --- | --- | --- | --- | --- | --- | --- | --- | --- | --- | --- | --- | --- | --- | --- | --- | --- | --- | --- | --- | --- |
| Indole | + | + | + | + | + | + | V | + | + | + | + | + | - | + | + | + | V | - | - | + | + | - | + | + | + | + | - | + | + | - |
| ONPG | + | + | + | + | + | V | + | + | - | + | + | + | + | + | + | + | + | nd | + | + | + | nd | + | + | + | + | + | - | +* | - |
| ADH | + | V | + | + | + | V | + | V | - | + | + | + | + | + | V | V | + | + | + | - | + | + | - | + | + | + | + | + | + | - |
| LDC | - | - | - | + | V | V | - | - | + | + | + | + | V | + | - | + | - | + | - | + | + | V | - | + | - | - | - | - | + | - |
| ODC | - | - | - | - | - | - | - | - | - | - | - | + | - | - | - | V | - | - | - | - | + | - | - | - | - | - | - | - | - | - |
| Citrate | - | - | - | + | - | + | + | - | + | + | V | + | V | + | - | V | + | nd | + | + | V | V | + | nd | + | - | V | nd | - | + |
| DL-lactate | - | + | V | V | - | - | + | - | - | - | - | - | V | + | - | - | - | nd | V | + | - | - | nd | - | nd | nd | - | - | - | - |
| H2S | - | - | - | + | V | V | - | V | + | V | V | - | - | - | V | V | + | nd | - | - | + | nd | nd | + | - | - | - | V | - | nd |
| VP | - | - | - | + | V | V | - | - | - | + | + | V | V | - | - | - | + | - | - | - | - | V | - | + | - | - | - | V | + | + |
| Gelatinase | V | V | V | + | V | + | V | + | - | + | + | + | V | + | - | + | + | nd | + | - | + | nd | - | + | + | + | + | + | + | + |
| D-Mannitol | + | + | + | + | + | + | + | + | + | + | + | + | - | V | + | + | + | - | + | + | + | + | - | + | + | + | + | - | - | + |
| Inositol | - | - | - | - | - | - | - | - | - | - | - | - | - | - | - | V | - | - | - | - | - | - | - | - | - | - | - | - | - | - |
| D-Sorbitol | - | - | - | - | - | + | - | - | - | - | - | - | - | - | - | - | - | - | - | - | - | - | - | V | - | - | - | - | - | - |
| L-rhamnose | - | - | - | V | V | - | - | V | - | - | - | - | - | - | V | V | - | - | - | - | - | - | - | nd | - | - | - | - | - | - |
| D-Saccharose | + | + | + | + | + | + | + | V | + | - | + | + | - | V | V | + | - | + | + | + | + | - | + | + | + | + | + | - | + | - |
| Amygdalin | V | + | - | - | - | - | - | - | - | - | - | - | - | - | - | - | - | - | nd | nd | nd | - | - | - | + | + | - | nd | - | - |
| L-arabinose | + | + | V | + | + | V | + | V | - | - | - | - | - | - | - | V | - | - | + | + | - | - | - | - | + | + | - | - | - | + |
| Cellobiose | + | + | + | - | V | V | + | V | + | - | V | V | - | + | - | + | - | + | V | + | - | - | + | - | - | - | V | - | - | - |
| Mannose | + | + | + | + | + | + | V | + | + | + | + | + | + | + | + | + | + | + | + | - | + | - | + | + | - | - | + | - | + | + |
| Pyrazinamidase | - | - | - | V | V | V | + | + | - | - | V | - | - | - | - | V | - | + | nd | nd | nd | nd | nd | nd | nd | nd | nd | nd | - | nd |
| Ammmonium chloride | V | + | V | nd | nd | nd | nd | nd | nd | nd | nd | nd | nd | nd | nd | nd | nd | nd | nd | nd | nd | nd | nd | nd | nd | nd | nd | nd | nd | nd |
| Lactose | V | V | V | + | - | + | V | - | - | - | - | V | - | - | - | - | - | - | - | - | - | - | + | - | - | - | - | - | - | - |
| Gas from glucose | V | V | V | + | V | V | - | V | V | + | + | + | - | V | V | + | + | - | - | - | + | + | + | + | - | - | - | - | - | - |

| Character | **A** | **B** | **C** | 28 | 29 | 30 |
| --- | --- | --- | --- | --- | --- | --- |
| Indole | + | + | + | + | + | V |
| ONPG | + | + | + | - | - | + |
| ADH | + | V | + | + | + | + |
| LDC | - | - | - | - | + | V |
| ODC | - | - | - | - | - | - |
| Citrate | - | - | - | - | + | + |
| DL-lactate | - | + | V | - | - | - |
| H2S | - | - | - | - | - | - |
| VP | - | - | - | - | - | + |
| Gelatinase | V | V | V | + | + | + |
| D-Mannitol | + | + | + | + | + | + |
| Inositol | - | - | - | - | V | - |
| D-Sorbitol | - | - | - | - | - | - |
| L-rhamnose | - | - | - | - | - | - |
| D-Saccharose | + | + | + | - | - | - |
| Amygdalin | V | + | - | - | - | - |
| L-arabinose | + | + | V | - | - | - |
| Cellobiose | + | + | + | + | + | + |
| Mannose | + | + | + | + | + | + |
| Pyrazinamidase | - | - | - | nd | nd | nd |
| Ammmonium chloride | V | + | V | nd | nd | nd |
| Lactose | V | V | V | + | - | - |
| Gas from glucose | V | V | V | + | + | + |

Taxa: A, *A. rivipollensis* (n = 20) except cellobiose/D-mannose/pyrazinamidase/ammonium chloride (n=4); B, *A. media* (n = 15) except cellobiose/D-mannose /pyrazinamidase /ammonium chloride (n=4); C, *Aeromonas* sp. genomospecies *paramedia* (n = 2); 1, *A. hydrophila* (n = 25); 2, *A. bestiarum* (n = 16); 3, *A. salmonicida* (n = 13); 4, *A. caviae* (n = 25); 5, *A. eucrenophila* (n = 9); 6, *A. sobria* (n = 2); 7, *A. jandaei* (n = 15); 8, *A. veronii* biovar *sobria* (n = 25); 9, *A. veronii* biovar *veronii* (n = 10); 10, *A. schubertii* (n = 12); 11, *A. enteropelogenes* (n = 16); 12, *A. encheleia* (n = 4); 13, *A. allosaccharophila* (n = 3); 14, *A. popoffii* (n = 7); 15, *A. simiae* (n = 1) (Harf-Monteil et al., 2004) tested at 30°C ; 16, *A. molluscorum* (n = 5) (Miñana-Galbis et al., 2004), tested at 25 ◦ C; 17, *A. bivalvium* (n = 2) (Miñana-Galbis et al., 2007), tested at 25–30 ◦ C; 18, *A. dhakensis* (n = 13) (Martínez-Murcia et al., 2008) tested at 30°C; 19, *A. tecta* (n = 5) (Demarta et al., 2008) tested at 30°C; 20, *A. fluvialis* (n = 1) tested at 30°C; 21, *A. piscicola* (n = 5) (Beaz-Hidalgo et al., 2009) tested at 25 ◦ C; 22, *A. taiwanensis* (n = 1) (Alperi et al., 2010) tested at 30°C; 23, *A. sanarellii* (n = 1) (Alperi et al., 2010) tested at 30°C; 24, A. rivuli (n = 2) (Figueras et al., 2011) tested at 30°C; 25, *A. diversa* (n = 2) (Miñana-Galbis et al., 2010) tested at 30°C; 26, *A. australiensis* (n = 1) (Aravena-Román et al., 2013); 27, *A. cavernicola* (n=1) (Martínez-Murcia et al., 2013) tested at 30°C ; 28, *A. aquatica* (n = 2) (Beaz-Hidalgo et al., 2015) tested at 30°C; 29, *A. finlandiensis* (n = 7) (Beaz-Hidalgo et al., 2015) tested at 30°C; 30, *A. lacus* (n = 2) (Beaz-Hidalgo et al., 2015) tested at 30°C. All tests were performed at 35°C. Data obtained from other studies tested at other temperatures is indicated. Data from 1 to 14 were obtained from Abbott et al. (Abbott et al., 2003) that performed the tests also at 35 °C with the exceptions of *A. popoffii* and *A. sobria* which were at 25 °C.

Abbreviations: +, 85–100% of strains positive; −, 0–15% of strains positive; V, 16–84% of strains positive; nd, not determined. VP, Voges-Proskauer.

*positive reaction when tested by disc (Rosco) but not in the API 20E strip.

**REFERENCES OF SUPPLEMENTARY TAXONOMIC SHEET**

Abbott, S. L., Cheung, W. K. W., and Janda, J. M. (2003). The genus Aeromonas: biochemical characteristics, atypical reactions, and phenotypic identification schemes. *J. Clin. Microbiol.* 41, 2348–2357.

Alperi, A., Martínez-Murcia, A. J., Ko, W.-C., Monera, A., Saavedra, M. J., and Figueras, M. J. (2010). Aeromonas taiwanensis sp. nov. and Aeromonas sanarellii sp. nov., clinical species from Taiwan. *Int. J. Syst. Evol. Microbiol.* 60, 2048–2055. doi:10.1099/ijs.0.014621-0.

Altwegg, M., Steigerwalt, A. G., Altwegg-Bissig, R., Lüthy-Hottenstein, J., and Brenner, D. J. (1990). Biochemical identification of Aeromonas genospecies isolated from humans. *J. Clin. Microbiol.* 28, 258–264.

Aravena-Román, M., Beaz-Hidalgo, R., Inglis, T. J. J., Riley, T. V., Martínez-Murcia, A. J., Chang, B. J., et al. (2013). Aeromonas australiensis sp. nov., isolated from irrigation water. *Int. J. Syst. Evol. Microbiol.* 63, 2270–2276. doi:10.1099/ijs.0.040162-0.

Beaz-Hidalgo, R., Alperi, A., Figueras, M. J., and Romalde, J. L. (2009). Aeromonas piscicola sp. nov., isolated from diseased fish. *Syst. Appl. Microbiol.* 32, 471–479. doi:10.1016/j.syapm.2009.06.004.

Beaz-Hidalgo, R., Latif-Eugenín, F., Hossain, M. J., Berg, K., Niemi, R. M., Rapala, J., et al. (2015). Aeromonas aquatica sp. nov., Aeromonas finlandiensis sp. nov. and Aeromonas lacus sp. nov. isolated from Finnish waters associated with cyanobacterial blooms. *Syst. Appl. Microbiol.* doi:10.1016/j.syapm.2015.02.005.

Demarta, A., Küpfer, M., Riegel, P., Harf-Monteil, C., Tonolla, M., Peduzzi, R., et al. (2008). Aeromonas tecta sp. nov., isolated from clinical and environmental sources. *Syst. Appl. Microbiol.* 31, 278–286. doi:10.1016/j.syapm.2008.04.005.

Figueras, M. J., Alperi, A., Beaz-Hidalgo, R., Stackebrandt, E., Brambilla, E., Monera, A., et al. (2011). Aeromonas rivuli sp. nov., isolated from the upstream region of a karst water rivulet. *Int. J. Syst. Evol. Microbiol.* 61, 242–248. doi:10.1099/ijs.0.016139-0.

Harf-Monteil, C., Flèche, A. L., Riegel, P., Prévost, G., Bermond, D., Grimont, P. A. D., et al. (2004). Aeromonas simiae sp. nov., isolated from monkey faeces. *Int. J. Syst. Evol. Microbiol.* 54, 481–485.

MacFaddin, J. (1993). *Pruebas bioquímicas para la identificación de bacterias de importancia clínica*. The William & William Company, Baltimore (Translation by Médica Panamericana S.S.).

Martínez-Murcia, A., Beaz-Hidalgo, R., Svec, P., Saavedra, M. J., Figueras, M. J., and Sedlacek, I. (2013). Aeromonas cavernicola sp. nov., isolated from fresh water of a brook in a cavern. *Curr. Microbiol.* 66, 197–204. doi:10.1007/s00284-012-0253-x.

Martínez-Murcia, A. J., Saavedra, M. J., Mota, V. R., Maier, T., Stackebrandt, E., and Cousin, S. (2008). Aeromonas aquariorum sp. nov., isolated from aquaria of ornamental fish. *Int. J. Syst. Evol. Microbiol.* 58, 1169–1175. doi:10.1099/ijs.0.65352-0.

Michon, A.-L., Jumas-Bilak, E., Chiron, R., Lamy, B., and Marchandin, H. (2014). Advances toward the elucidation of hypertonic saline effects on Pseudomonas aeruginosa from cystic fibrosis patients. *PloS One* 9, e90164. doi:10.1371/journal.pone.0090164.

Miñana-Galbis, D., Farfán, M., Fusté, M. C., and Lorén, J. G. (2004). Aeromonas molluscorum sp. nov., isolated from bivalve molluscs. *Int. J. Syst. Evol. Microbiol.* 54, 2073–2078. doi:10.1099/ijs.0.63202-0.

Miñana-Galbis, D., Farfán, M., Fusté, M. C., and Lorén, J. G. (2007). Aeromonas bivalvium sp. nov., isolated from bivalve molluscs. *Int. J. Syst. Evol. Microbiol.* 57, 582–587. doi:10.1099/ijs.0.64497-0.

Miñana-Galbis, D., Farfán, M., Gaspar Lorén, J., and Carmen Fusté, M. (2010). Proposal to assign Aeromonas diversa sp. nov. as a novel species designation for Aeromonas group 501. *Syst. Appl. Microbiol.* 33, 15–19. doi:10.1016/j.syapm.2009.11.002.
