# Supplementary material for: Delineation of Taxonomic Species within Complex of Species: Aeromonas media and Related Species as a Test Case
Source: Front Microbiol. 2017 Apr 18;8:621. doi: 10.3389/fmicb.2017.00621 (PMC5394120; doi:10.3389/fmicb.2017.00621)
Supplement: Supplementary file 5 [file DataSheet2.DOCX]

**Supplementary Figure 1: Unrooted Maximum-Likelihood tree based on 16S rRNA sequences (1,322 bp) showing the relationships between strains affiliated to *Media* species complex, with other type strains of all *Aeromonas* species.** The sequences of the strains affiliated to the *Media* species complex were obtained from consensus 16S rRNA sequences between the different copies, except for ‘*A. hydrophila’* 4AK4, *A. media* WS and *A. rivipollensis* P2G1^T^ for which the 16S rRNA sequence data were extracted from public databases. The horizontal lines represent genetic distance, with the scale bar indicating the number of substitutions per nucleotide position. The numbers at the nodes are support values estimated with 100 bootstrap replicates. Only bootstrap values ≥50 are indicated.

MLP: Multi-Locus Phylogeny
